# Using the Grey Wolf Aquila Synergistic Algorithm for Design Problems in Structural Engineering

**DOI:** 10.3390/biomimetics9010054

**Published:** 2024-01-18

**Authors:** Megha Varshney, Pravesh Kumar, Musrrat Ali, Yonis Gulzar

**Affiliations:** 1 Rajkiya Engineering College, Dr. APJ Abdul Kalam Kalam Technical University, Bijnor 246725, India; 2 Department of Basic Sciences, General Administration of Preparatory Year, King Faisal University, Al-Ahsa 31982, Saudi Arabia; 3 Department of Management Information Systems, College of Business Administration, King Faisal University, Al-Ahsa 31982, Saudi Arabia; ygulzar@kfu.edu.sa

**Keywords:** Aquila Optimizer, grey wolf optimization, quasi-opposition-based learning, real-world engineering problems

## Abstract

The Aquila Optimizer (AO) is a metaheuristic algorithm that is inspired by the hunting behavior of the Aquila bird. The AO approach has been proven to perform effectively on a range of benchmark optimization issues. However, the AO algorithm may suffer from limited exploration ability in specific situations. To increase the exploration ability of the AO algorithm, this work offers a hybrid approach that employs the alpha position of the Grey Wolf Optimizer (GWO) to drive the search process of the AO algorithm. At the same time, we applied the quasi-opposition-based learning (QOBL) strategy in each phase of the Aquila Optimizer algorithm. This strategy develops quasi-oppositional solutions to current solutions. The quasi-oppositional solutions are then utilized to direct the search phase of the AO algorithm. The GWO method is also notable for its resistance to noise. This means that it can perform effectively even when the objective function is noisy. The AO algorithm, on the other hand, may be sensitive to noise. By integrating the GWO approach into the AO algorithm, we can strengthen its robustness to noise, and hence, improve its performance in real-world issues. In order to evaluate the effectiveness of the technique, the algorithm was benchmarked on 23 well-known test functions and CEC2017 test functions and compared with other popular metaheuristic algorithms. The findings demonstrate that our proposed method has excellent efficacy. Finally, it was applied to five practical engineering issues, and the results showed that the technique is suitable for tough problems with uncertain search spaces.

## 1. Introduction

In order to maximize profit, productivity, and efficiency, optimization is carried out, which is basically the process of identifying the best possible solution among all viable options for a particular situation [1,2,3]. In recent decades, as human culture and contemporary science have advanced, the intricacy of the number of optimization issues in the actual world has been rising significantly, placing more demands on optimization strategies’ dependability and efficacy [4]. In general, deterministic algorithms and metaheuristic algorithms (MAs) are two categories of existing optimization technologies that are used. Using the same starting parameters for a deterministic method, possible solutions are produced in accordance with mechanical convergence to the global optimum without regard to the analytical features of problems or anything arbitrary [5]. The conjugate gradient and the Newton–Raphson technique are two typical deterministic techniques. While this kind of method can solve some nonlinear problems satisfactorily, it often falls into local optima when met with multimodal, large-scale, and sub-optimal search space constraints. It also requires the problem’s derivative information. Lately, as a perfect substitute for deterministic algorithms, MAs are gaining popularity among academics worldwide as a great substitute for deterministic algorithms because of their straightforward designs, minimal processing overheads, ability to avoid gradient information, and strong local optimal avoidance capability [6].

Metaheuristic algorithms [7] are influenced by nature [8]. Based on their sources of inspiration, these algorithms can be grouped into four categories [9,10], including Evolution-based Algorithms (EAs) [11], Swarm-based Intelligence (SI) [12], Physics-based Techniques (PTs) [13], and Human-based Behaviors (HBs) [14], as indicated in Table 1. They usually model physical or biological phenomena in nature and create mathematical frameworks to solve optimization problems [15,16]. These algorithms offer the features of self-organization, self-adaptation, and self-learning, and they have been widely applied in various domains, such as biology [17,18], feature selection [19], optimization computing [20], image classification [21], and artificial intelligence [22,23].

Despite Metaheuristic Algorithms’ (MAs’) success in many areas of computational research, they may nevertheless experience a poor convergence speed, a propensity to converge too early, and a tendency to fall into local optima [42].

The No-Free-Lunch (NFL) theorem [43] states that no single algorithm can solve all optimization problems. Because of this theorem, many academics devote their time to creating new MAs or improving old ones. In addition to adding certain efficient search techniques, it has recently been fashionable to combine the two fundamental MAs for a more effective overall performance when enhancing existing algorithms. As opposed to a single algorithm, a hybrid algorithm encourages diversity and spreads more helpful information throughout its population, giving it a stronger search power.

In this study, we concentrate on the two most recent swarm-based MAs, namely the Aquila Optimizer and Grey Wolf Optimizer. Concurrently, the improved initialization approach employs the quasi-oppositional-based learning (QOBL) [44] strategy to produce an opposing solution that guides the AO algorithm’s search phase.

The Aquila Optimizer (AO) was first proposed in 2021 [29]. It can find solutions with a certain level of precision at a cheap computational cost, it is easy to implement, and it requires little fine-tuning of its parameters. Thus, it has excellent research potential. For people who are new to metaheuristic optimization, this makes it a good option. The Aquila Optimizer has undergone numerous improvements to make it more potent at resolving challenging real-world optimization issues; some of the recent improvements are hybridization NIOAs [45,46], opposition-based learning (OBL) [47], quasi-oppositional-based learning (QOBL) [48], Chaotic Sequence [49], Levy-flight-based strategy [50], Gauss map and crisscross operator [51], random learning mechanism and Nelder–Mead simplex search [52], wavelet mutation [53], weighted adaptive searching technique [54], binary AO [55], etc. A fine literature examination of the AO algorithm and its application is offered in reference [56]. From these sources, it may be inferred that AO has a propensity to converge too soon and undergo local stagnation.

The other method concerned in this paper, the GWO, was created in 2014 [30]. The Grey Wolf Optimizer (GWO) was developed in response to the social hunting behavior of grey wolves and has inspired other academics to use it to tackle practical optimization issues. Grey wolves have a rigid social structure with an alpha wolf at the top, and they live in packs. The alpha wolf is in charge of steering the pack and making choices, and the other wolves adhere to its instructions [57]. The top solution thus far has been identified to be the alpha position of the GWO. So, we may force the AO to search through fresh regions of the search space and prevent local stagnation by adding the alpha position of the GWO into the AO.

Opposition-based learning (OBL) [58] is a novel area of study that has generated noteworthy attention within the past 10 years. By making use of the OBL concept, numerous soft computing methods have been improved. To boost the solution quality, the quasi-opposition-based learning (QOBL) technique [59] is applied. The QOBL indicates that, when solving an optimization issue, it is more effective to employ a quasi-opposite number than an opposite number. The fittest population in QOBL is made up of the current nominee population as well as its opposite population and quasi-opposite population. The QOBL strategy can be applied in integrating NIOAs, in the optimal design of PI/PD dual-mode controllers [60], and in the parameter identification of permanent magnet synchronous motors [61].

In light of the above discussion, to increase the exploration ability of the AO algorithm, this work offers a hybrid approach that employs the alpha position of the GWO to drive the search process of the AO algorithm. At the same time, we applied the QOBL strategy in each phase of the Aquila Optimizer. This strategy develops quasi-oppositional solutions to the current solutions. The quasi-oppositional solutions are then utilized to direct the search phase of the AO algorithm. We call this enhanced algorithm the GAOA. By comparing the application results of 10 swarm intelligence algorithms based on 23 classical benchmark functions [29,30] and 29 CEC2017 benchmark functions [62], it is proven that the approach suggested in this research can speed up the convergence speed, enhance the convergence accuracy, and identify the global optimum instead of the local optimum. The application of four engineering challenges also indicates that our suggested approach has considerable advantages in solving genuine situations.

The important contributions of this study are summarized as follows:

- Based on the Grey Wolf alpha position, the Aquila Optimizer has been improved, so that its exploration ability is increased.
- Then, the quasi-oppositional-based learning strategy is used in each phase of the Aquila Optimizer to direct the search process of the AO algorithm.
- The performance of our method on 23 classical functions and 29 CEC2017 functions is examined and compared with the performances of the other 10 algorithms while considering different dimensions.
- Four engineering design challenges are utilized to evaluate the effectiveness of our proposed method to solve practical situations.

The following chapters of this article are organized as follows: Section 2 introduces the background of the Aquila Optimizer, Grey Wolf Optimizer, and opposition-based learning strategy. Section 3 introduces the developed algorithm. In Section 4, we carry out the corresponding experiment. Also, four traditional engineering problems are discussed in Section 5. Finally, Section 6 provides a summary and the future prospects.

## 2. Background

### 2.1. Aquila Optimizer

The metaheuristic optimization method, known as the Aquila Optimizer (AO) [29], was motivated by the Aquila bird’s hunting style. The Aquila bird uses four primary prey hunting techniques, which AO imitates: Using its height advantage, the Aquila bird swoops down vertically to take down floating prey. By hovering in a contour-like pattern close to the ground, the Aquila bird performs swift glide-like assaults to pursue seabirds. The Aquila bird uses this technique to hunt foxes and other prey that move slowly. The Aquila bird employs this method to directly capture prey while walking on the ground.

#### 2.1.1. Expanded Exploration

The Aquila Optimizer algorithm’s expanded exploration $u_{1}$ reflects that it achieves great heights, and then descends rapidly, which is the hunting method observed in Aquila birds. This tactic involves the bird soaring at great heights, allowing it to thoroughly examine the search area, spot prospective prey, and choose the best hunting location. Ref. [29] contains a mathematical representation of this tactic, as shown in Equation (1).

(1) $$u_{1}^{\left(h+1\right)}=u_{best}^{\left(h\right)}\times \left(1-\frac{h}{H}\right)+\left(u_{M}^{\left(h\right)}-rand\times u_{best}^{\left(h\right)}\right)$$

The maximum number of iterations in the method is symbolized as H whereas h stands for the present iteration. The initial search in the candidate solution population $\left(u_{1}\right)$ yields the answer for the following iteration, denoted as $\left(u_{1}^{\left(h+1\right)}\right)$. The expression $\left(u_{best}^{\left(h\right)}\right)$ denotes the best result up to the $h^{th}$ iteration. Through the equation $\left(1-\frac{h}{H}\right)$, to adjust the depth of the search space, a count of iterations is used. Furthermore, N denotes the population size and dimension size, D, and the average value of the locations of connected existing solutions at the $h^{th}$ iteration is calculated using Equation (2), indicated as $u_{M}^{\left(h\right)}$.

(2) $$u_{M}^{\left(h\right)}=\frac{1}{N}\sum_{i=1}^{N}u_{i}\left(h\right),\;\mathrm{for}\;\mathrm{all}\;j=1,2,\ldots ,D$$

#### 2.1.2. Narrowed Exploration

The Aquila Optimizer algorithm’s narrowed exploration method $\left(u_{2}\right)$ is in line with how Aquila birds hunt; to pursue prey, the tactic entails flying in a contour-like pattern with quick gliding attacks in a condensed investigation area. The main objective of this approach, which is mathematically described in Equation (3), is to find a solution $\left(u_{2}^{\left(h+1\right)}\right)$ for the following iterations, denoted as h.

(3) $$u_{2}^{\left(h+1\right)}=u_{best}^{\left(h\right)}\times Levy\left(D\right)+u_{R}^{\left(h\right)}+\left(v-u\right)\times rand$$

The Levy flying distribution for dimension space, D, is referred to as Levy(D) in the Aquila optimization method. In the range [1,N], where N denotes population size, the random solution $\left(u_{R}^{\left(h\right)}\right)$ is discovered at the $h^{th}$ iteration. Typically set to 0.01, the fixed constant value s is used to determine the Levy flight distribution along with randomly chosen parameters, u and v, that range from 0 to 1. Equation (4) provides the mathematical expression for this calculation.

(4) $$Levy\left(D\right)=s\times \frac{u\times \sigma }{{\left|v\right|}^{\frac{1}{\alpha }}}$$

Equation (5) determines the value s, which is derived using a certain constant parameter a set at 1.5.

(5) $$\sigma =\left(\frac{\gamma \left(1+a\right)\times \sin\left(\frac{\pi a}{2}\right)}{\gamma \left(\frac{\left(1+a\right)}{2}\right)\times a\times 2^{\left(\frac{a-1}{2}\right)}}\right)$$

The spiral shapes within the search range, designated by v and u, respectively, are represented by Equations (6) and (7). These spiral shapes are specified in Equation (3).

(6) $$v=r_{1}+UD_{1}\cos\left(-\omega D_{1}+\left(\frac{3\pi }{2}\right)\right)$$

(7) $$u=r_{1}+UD_{1}\sin\left(-\omega D_{1}+\left(\frac{3\pi }{2}\right)\right)$$

Over a predetermined number of search iterations, the variable $r_{1}$ accepts values in the range of (1, 20). w and U have fixed values of 0.005 and 0.00565, respectively. $D_{1}\in \mathbb{Z}$ has a range of 1 to the search space’s dimension, D.

#### 2.1.3. Expanded Exploitation

The Aquila bird targets its prey with a low, slow-moving descent attack while carefully inspecting the prey’s location during the investigation phase. Equation (8) is a mathematical representation of this method, also known as expanded exploitation $u_{3}$.

(8) $$u_{3}^{\left(h+1\right)}=\left(u_{best}^{h}-u_{M}^{\left(h\right)}\right)\times \theta -rand+\left(\left(ub-lb\right)\times rand+lb\right)\times \rho$$

Equation (8) results in $\left(u_{3}^{\left(h+1\right)}\right)$, which denotes the outcome for the following iteration. In the $h^{th}$ iteration, $\left(u_{M}^{\left(h\right)}\right)$ stands for the average value of the current solution determined by Equation (2), and $u_{best}\left(h\right)$ represents the currently best solution found. The tuning parameters θ and ρ are normally given a value of 0.1 each, whereas the variable ‘rand’ is allocated a random number in the (0, 1) range. The upper bound is shown as ub, and the lower bound is shown as lb.

#### 2.1.4. Narrowed Exploitation

Aquila birds use a hunting strategy in which they directly capture their targets by exploiting the prey’s erratic movement patterns when on the ground. Equation (9), which generates the $h^{th}$ iteration of the following solution, indicated as $\left(u_{4}^{\left(h+1\right)}\right)$, uses this hunting approach as the foundation for the restricted exploitation technique $\left(u_{4}^{\left(h\right)}\right)$ design. A quality function known as J, which is stated in Equation (10), was proposed to guarantee a balanced search strategy.

(9) $$u_{4}^{\left(h+1\right)}=J\times u_{best}^{\left(h\right)}-\left(P_{1}\times rand\times u_{1}^{\left(h\right)}\right)-P_{2}\times Levy\left(D\right)+rand\times P_{1}$$

Equations (11) and (12) are utilized to calculate the trajectory of an attack during a getaway, from the initial location to the terminal location $\left(P_{2}\right)$, and the motion pattern for the Aquila bird’s prey tracking $\left(P_{1}\right)$. The calculations are performed using the current iteration number (h) and the maximum number of iterations (H).

(10) $$J\left(h\right)=h^{\frac{2\times rand()-1}{{\left(1-H\right)}^{2}}}$$

(11) $$P_{1}=2\times rand-1$$

(12) $$P_{2}=2\times \left(1-\frac{h}{H}\right)$$

The pseudocode in Algorithm 1 provides a summary of the Aquila Optimization procedure.
**Algorithm 1** Aquila OptimizerSet Initial values of parameters (nPop, nVar, α, β, max_iter, etc.) where nPop refers to population size, max_iter to the maximum number of iterations. Determine the starting position at random. While (Iteration < max_iter) do Determine the fitness of early positions. As $u_{best}\left(h\right)$, identify the best individual with the finest fitness values. For (i = 1: nPop)  Updated variables include $u,\;v,\;P_{1},\;P_{2},\;and\;Levy\left(D\right)$  If $h\leq \left(\frac{2}{3}\right)\times H$ then   If rand≤0.5    Execute Expanded Exploration using Equation (1)   Else    Execute Narrowed Exploration using Equation (3)   End  Else   If rand≤0.5    Execute Expanded Exploitation using Equation (8)   Else    Execute Narrowed Exploitation using Equation (9)   End If  End If End for End while Record best solution $\left(u_{best}\right)$

### 2.2. Grey Wolf Optimizer

The GWO is a population-based metaheuristic algorithm [30] that replicates grey wolves, considered as apex predators, which are at the top of the food chain [57]:

- The alpha wolf is regarded as the dominating wolf in the pack, and his/her orders should be followed by the pack members.
- Beta wolves are subordinate wolves, which support the alpha wolf in decision making, and they are considered the best prospects to be the alpha wolf.
- Delta wolves have to surrender to the alpha and beta, but they rule the omega.
- Omega wolves are regarded as the scapegoats in the pack, they are the least important individuals in the pack, and they are only allowed to feed last.

#### 2.2.1. Encircling the Prey

When the prey site is seized by the grey wolves, the encircling of prey is performed. In the process of encircling, grey wolf individuals should first assess the distances between themselves and the prey according to Equation (13) and then update their positions through Equation (14):

(13) $$\vec{D}=\left|\vec{C}\cdot u_{p}\left(h\right)-u\left(h\right)\right|$$

(14) $$u\left(h+1\right)=u_{p}\left(h\right)-\vec{A}\cdot \vec{D}$$

where h denotes the current iteration, $\vec{A}$ and $\vec{C}$ are specified as coefficient vectors, $u_{p}$ represents the best solution position vector that the prey has detected so far, and u indicates the position vector of a grey wolf. $\vec{D}$ is the difference vector that chooses the movement of the wolf either toward the neighborhood areas of the prey or opposite of them.

Both $\vec{A}$ and $\vec{C}$ are modified over iterations like the following:

(15) $$\vec{C}=2{\vec{r}}_{2}$$

(16) $$\vec{A}=2\vec{a}\cdot {\vec{r}}_{1}-\vec{a}$$

where ${\vec{r}}_{1}$ and ${\vec{r}}_{2}$ are randomly generated stochastic vectors from the interval [0, 1]. $\vec{C}$ and $\vec{A}$ are coefficients that are determined by Equations (15) and (16). The components of the vector $\vec{a}$ are linearly decreasing from 2 to 0 during the course of the iterations and can be stated as Equation (17):

(17) $$a=2-2\times \frac{h}{H}$$

where h signifies the current iteration, and H denotes the maximum number of iterations.

#### 2.2.2. Hunting the Prey

In the GWO, while the global optimums of an optimization problem are unknown, the first three grey wolves, the alpha, beta, and delta, are always assumed to be the closest solutions to the optimal value. In the hunting strategy, the placements of each search agent (wolf) are altered based on the three best places of the alpha, beta, and delta. The following equations are used to replicate the hunting process and to locate the better optimum in the border space. Therefore, the remaining wolves are required to update their positions following the leading wolves, which may be computed by Equations (18)–(20).

(18) $$\begin{array}{c} {\vec{D}}_{\alpha }=\left|{\vec{C}}_{1}\cdot u_{\alpha }\left(h\right)-u\left(h\right)\right|\\ {\vec{D}}_{\beta }=\left|{\vec{C}}_{1}\cdot u_{\beta }\left(h\right)-u\left(h\right)\right|\\ {\vec{D}}_{\delta }=\left|{\vec{C}}_{1}\cdot u_{\delta }\left(h\right)-u\left(h\right)\right| \end{array}$$

(19) $$\begin{array}{l} u_{1}=u_{\alpha }-{\vec{A}}_{1}\cdot \left({\vec{D}}_{\alpha }\right)\\ u_{2}=u_{\beta }-{\vec{A}}_{2}\cdot \left({\vec{D}}_{\beta }\right)\\ u_{3}=u_{\delta }-{\vec{A}}_{3}\cdot \left({\vec{D}}_{\delta }\right) \end{array}$$

(20) $$u\left(h+1\right)=\frac{u_{1}+u_{2}+u_{3}}{3}$$

where $u_{\alpha }$, $u_{\beta }$, and $u_{\delta }$ are the three best positions of the alpha, beta, and delta; ${\vec{D}}_{\alpha }$, ${\vec{D}}_{\beta }$, and ${\vec{D}}_{\delta }$ are the distances of the search agents away from the three best solutions; and ${\vec{A}}_{1}$, ${\vec{A}}_{2}$, and ${\vec{A}}_{3}$ represent random vectors.

#### 2.2.3. Attacking the Prey (Exploitation Phase)

Grey wolves separate from each other to look for prey and converge to attack prey. Grey wolves will only attack the prey when they are no longer moving. This phase is responsible for exploitation and is handled by a linear decrement in $\vec{a}$.

The linear reduction in this parameter enables grey wolves to attack the prey when it stops moving.

#### 2.2.4. Searching the Prey (Exploration Phase)

It is apparent that after the prey stops moving, the wolf will kill the prey and, in this way, they finish their hunting process. Grey wolves primarily search according to the positions of α, β, and δ [63].

The process of the GWO can be exhibited in detail according to the pseudo-code of the GWO’s Algorithm 2.
**Algorithm 2** Grey Wolf OptimizerSet Initial values of parameters (nPop, max_iter, ub, and lb) Use ub and lb to generate the starting locations for the grey wolves. Initialize a=2,A,C Calculate each grey wolf’s fitness level. The grey wolf with the highest level of fitness is the $u_{\alpha }$. The grey wolf with the second-highest fitness level is $u_{\beta }$. The grey wolf with the third highest fitness is called $u_{\delta }$. While (Iteration < max iteration)  for (i = 1: nPop) do     Report the current location of the grey wolf by using Equation (20)  end for Update $u_{\alpha }$, $u_{\beta }$, $u_{\delta }$. Calculate each search agent’s fitness. Return $u_{\alpha }$ while updating $u_{\beta }$, $u_{\delta }$.

The best answer discovered thus far in the search space is represented by the alpha wolf. It increases the speed and efficiency of convergence by guiding the other wolves, or candidate solutions, toward areas of potential interest. By doing this, early convergence to possibly less-than-ideal local optima is avoided. It promotes equilibrium behavior between exploration and exploitation. Compared to certain other metaheuristics, the algorithm is comparatively simple to develop and comprehend due to the simplicity of the alpha position notion. Therefore, for better performance, the alpha position can be combined with other optimization strategies to create flexible hybrid algorithms [64].

### 2.3. Opposition-Based Learning and Quasi-Opposition-Based Learning

Tizhoosh first proposed opposition-based learning (OBL) in 2005 [58]. By contrasting the current solution with the opposition-based learning solution, OBL’s primary goal is to select the best solution for the following iteration. Numerous metaheuristic algorithms have effectively employed the OBL approach to increase their ability to overcome the stagnation of local optima [65,66]. The following is the mathematical equation:

$$u_{OBL}\left(h+1\right)=lb+ub-u\left(h\right)$$

A better OBL variant is quasi-opposition-based learning (QOBL) [48], which uses quasi-opposite points rather than opposite points. QOBL points are more likely to represent challenging problems that have not yet been solved by existing methods. The QOBL mathematical equation is as follows:

$$u_{QOBL}\left(h+1\right)=\{\begin{array}{c} CS+r_{9}\times \left(MP-CS\right),\;\mathrm{if}\;MP>CS\\ MP+r_{9}\times \left(CS-MP\right),\;otherwise, \end{array}$$

$$CS=\frac{lb+ub}{2}$$

MP=lb+ub−u(h)

Below is the pseudo-code of implementing QOBL in population initialization, denoted as Algorithm 3.
**Algorithm 3** Quasi-Oppositional-Based LearningSet Initial values of parameters (nPop, nVar, initial population u, lb, ub) For i = 1: nPop For j = 1: nVar    $u_{i,j}^{o}=lb_{j}+ub_{j}-u_{i,j}$   %Inverting the current population   $D_{i,j}=\frac{lb_{j}+ub_{j}}{2}$  If $\left(u_{i,j}<D_{i,j}\right)$      %Creating Quasi Opposite of Population   $u_{i,j}^{qo}=D_{i,j}+\left(u_{i,j}^{o}-D_{i,j}\right)\times rand()$  Else    $u_{i,j}^{qo}=u_{i,j}+\left(D_{i,j}-u_{i,j}^{o}\right)\times rand()$  End End End

## 3. Proposed Framework

In this section, the general framework of the developed algorithm is described in Algorithm 4.

Using the GWO method to populate the AO algorithm’s initial population will boost its exploratory capabilities. To carry this out, a population of solutions can first be generated using the GWO algorithm. 

In Algorithm 4, once the population of the AO algorithm has been initialized, the AO algorithm can be utilized to optimize the issue. The alpha position of the GWO population can be utilized to steer the search process of the AO algorithm. This can be achieved by updating the positions of the solutions in the AO population based on the alpha position. To create an improved harmony between diversity and amplification and to make sure that the optimal result was found, the QOBL was reimplemented for each phase of the AO. Up until the termination criteria were satisfied, this process was repeated. We named this hybrid algorithm the Grey Wolf Aquila Synergistic Algorithm (GAOA).
**Algorithm 4** GAOA Set Initial values of parameters (nPop, max_iter, ub, and lb, nVar, α, β) Initialize Population Randomly While (Iteration < Max iterations) do Determine the fitness of each wolf. Evaluate the alpha position by using the GWO algorithm If $h\leq \left(\frac{2}{3}\right)\times H$  If rand≤0.5   Execute Expanded Exploration by using alpha position in Equation (1)   Execute QOBL  Else   Execute Narrowed Exploration by using alpha position in Equation (3)   Execute QOBL   End If Else  If rand≤0.5   Execute Expanded Exploitation by using alpha position in Equation (8)   Execute QOBL  Else   Execute Narrowed Exploitation by using the alpha position in Equation (9)   Execute QOBL  End If  End If End while Record best solution $\left(u_{best}\right)$

The performance of the AO algorithm can be enhanced by initializing the population of the AO algorithm with the GWO algorithm and directing the search process using the alpha position of the AO population.

**Figure 1:**
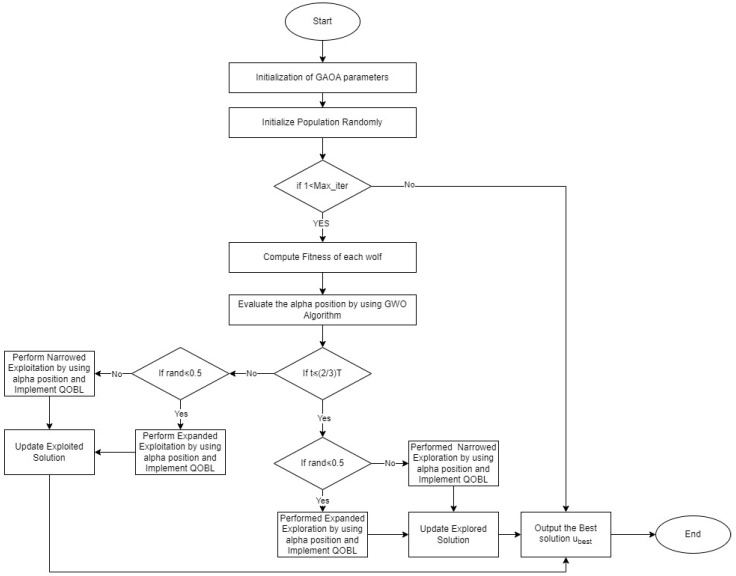
Flowchart of Grey Wolf Aquila Synergistic Algorithm (GAOA).

This is due to the AO algorithm’s potential to increase the likelihood of obtaining the global optimum, expand the search space under consideration, and decrease the likelihood of early convergence. Additionally, the harmony between diversity and intensification can be enhanced by including the quasi-oppositional-based learning (QOBL) technique in each stage of the AO algorithm. This may help the AO algorithm to perform even better. Its flowchart is given in Figure 1 for a clear visualization of the process.

Overall, the suggested method, the Grey Wolf Aquila Synergistic Algorithm (GAOA), is a promising strategy to enhance the AO algorithm’s performance in a variety of optimization tasks. It has been demonstrated to be successful at enhancing the performance of the AO method in a number of benchmark optimization tasks whilst being very easy to implement.

The general computational complexity of the GAOA is also shown in this section. Three rules are usually used to determine the computational complexity of the GAOA: initializing the solutions, calculating the fitness functions, and updating the solutions. 

Let N be the number of solutions and let O(N) be the computing complexity of the initialization procedures of the solutions. The updating processes for the solutions have a computational complexity of O(N×D)+O(G×(N×D+N×D)), where G is the total number of iterations and D is the problem’s dimension size. These processes involve searching for the best positions and updating each solution’s position. As a result, the suggested GAOA’s (Grey Wolf Aquila Synergistic Algorithm) overall computational complexity is O(N×D)+O(G×(N×D+N×D))=O(ND(1+2G)).

## 4. Experimental Results and Analysis

### 4.1. Experimental Settings

The performance of the suggested approach is examined in this work by utilizing benchmark functions from the IEEE Congress on Evolutionary Computation 2017 (CEC2017) and 23 classical benchmark functions. The test suite for the IEEE CEC2017 has 30 functions, although F2 is excluded due to instability. There are two unimodal functions (F1 and F3), seven basic multimodal functions (F4–F10), ten hybrid functions (F11–F20), and ten composition functions (F21–F30) among the twenty-nine benchmark functions.

The population size (N) was fixed at 100 in each experiment. The [−100, 100] range was chosen for the search. On each function, each algorithm was executed 51 times. In the tables that follow, the best results across all comparing algorithms are highlighted in bold. On a computer with an IntelI CoITM) i7-9750H processor running at 2.60 GHz and 16 GB of RAM, all algorithms were implemented in MATLAB R2021b.

**The following four factors are used to assess GAOA’s (Grey Wolf Aquila Synergistic Algorithm) performance:**

- The average and standard deviation of the optimization errors between the obtained and known real optimal values are used. All objective functions are minimization issues; hence, the best values, or minimum mean values, are denoted in bold.
- Non-parametric statistical tests, such as the Wilcoxon rank-sum test, are used to compare the *p*-value and the significance level (0.05) between the suggested algorithm and the compared method [67,68]. There is a substantial difference between the two algorithms when the *p*-value is less than 0.05. W/T/L denotes the number of wins, ties, and losses the given algorithm has experienced in comparison to its rival.
- Another non-parametric statistical test that is employed is the Friedman test [69]. As test data, the average optimization error values are employed. The algorithm performs better when the Friedman rank value is lower. The minimum value is bolded to draw attention to it.
- By exhibiting the pairwise variations in the ranks for each method at each dimension, the Bonferroni–Dunn diagram demonstrates the discrepancies between the rankings achieved for each algorithm at dimensions of 30, 50, and 100. By deducting the rank of one algorithm from the rank of another algorithm, the pairwise differences in the rankings are determined. Each bar in the Bonferroni–Dunn image represents the average pairwise difference in ranks for a particular algorithm at a particular dimension. Usually, the bars are color-coded to represent various algorithms.
- Convergence graphs are used to provide a simple visual representation of the algorithm’s accuracy and speed of convergence. It explains if the enhanced algorithm breaks away from the local solution.

### 4.2. Competitive Algorithms Comparison

Seven competing algorithms are compared to gauge the GAOA’s efficacy and search performance: the MAO (Modified Aquila Optimizer) [70], AO (Aquila Optimizer), GWO (Grey Wolf Optimizer), SCA (Sine–Cosine Algorithm), RSA (Reptile Search Algorithm), WOA (Whale Optimization Algorithm), SSA (Salp Swarm Algorithm). The comparison is made on 29 benchmark functions from IEEE CEC2017 with dimensions of 30, 50, and 100.

Table 2 displays the parameter settings [71] for various methods. Table 3, Table 4, Table 5 and Table 6 show the findings of a comparative experiment.

In Table 3, the GAOA, MAO, AO, GWO, SCA, RSA, WOA, and SSA attained the best mean values on 25, 1, 0, 2, 0, 0, 0, and 0 functions, respectively. The W/T/L metric shows that the GAOA performs well on functions with 30 dimensions, out-performing the MAO, AO, GWO, SCA, RSA, WOA, and SSA on 28, 29, 27, 29, 29, 29, and 27 functions, respectively.

In Table 4, it can be seen that the GAOA earned the best mean values on 21, 3, 0, 2, 0, 0, and 1 function, respectively. The W/T/L metric shows that the GAOA performs well on functions with 50 dimensions, outperforming the MAO, AO, GWO, SCA, RSA, WOA, and SSA on 21, 29, 25, 29, 29, 29, and 28 functions, respectively.

In Table 5, it can be seen that the GAOA earned the best mean values on 19, 5, 0, 4, 0, 0, and 1 function, respectively. The W/T/L metric shows that the GAOA performs well on functions with 100 dimensions, outperforming the MAO, AO, GWO, SCA, RSA, WOA, and SSA on 21, 29, 24, 28, 29, 28, and 27 functions, respectively.

To test the exploration, exploitation, and stagnation process avoidance abilities of the GAOA, a set of 23 benchmark test functions is employed. As seen in Table 6, the GAOA outperforms the SMA, SSA, and other metaheuristic algorithms by a significant margin. With the exception of F6, the GAOA, in particular, routinely beats the other algorithms. Notably, for all unimodal functions other than F5, the GAOA has the least mean value and standard deviations and achieves the theoretical optimum for F1–F4. These results show good precision and stability, emphasizing the excellent applicability of the suggested GAOA algorithm. The results for functions F8–F23 shown in Table 6 show that the GAOA also performs exceptionally well in exploration. The theoretical optimum is notably achieved by the GAOA in F8, F10, F14–F17, and F19–F23, highlighting its outstanding exploration capacity. These results demonstrate the strength of the GAOA in navigating the search space and locating the best answers.

Table 7’s Friedman test findings further demonstrate the GAOA’s better performance. The table shows that the IEEE CEC2017 functions with 30, 50, and 100 dimensions are the ones where the GAOA works best.

The statistical findings for each of the nine functions (F1, F3, F5, F6, F7, F9, F11, F12, and F13) are shown in Table 8 for each parameter setting. It is evident from these findings that, out of all the functions evaluated, the parameters of α=0.1 and δ=0.1 generally perform better in different circumstances, followed by α=0.1 and δ=0.5; α=0.1 and δ=0.9; α=0.5 and δ=0.1; and α=0.9 and δ=0.9, which assigned ranks 2, 3, 5, and 4, respectively. But the AO performs similarly in each of these instances at F1, F3, F9, and F11.

**Figure 2:**
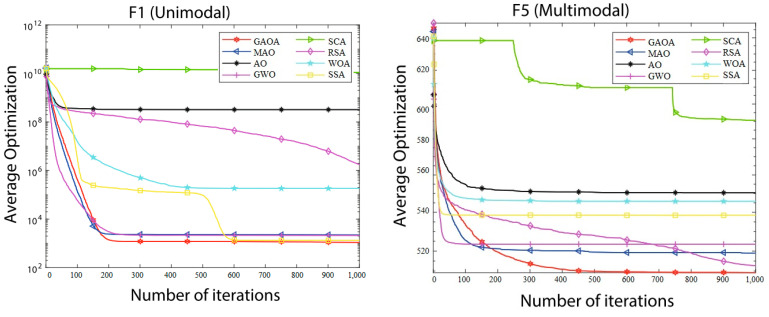

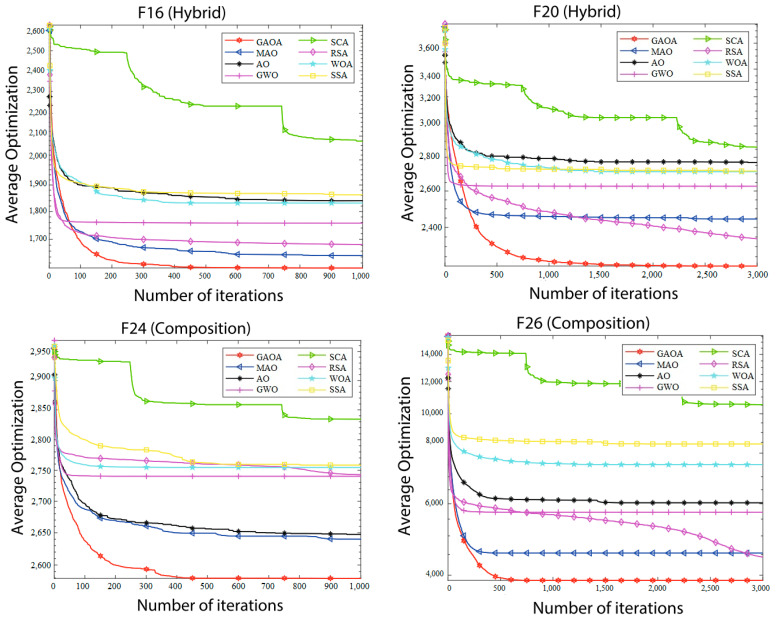
Convergence graphs of average optimizations obtained by 8 algorithms from CEC2017 functions (D = 30).

**Figure 3:**
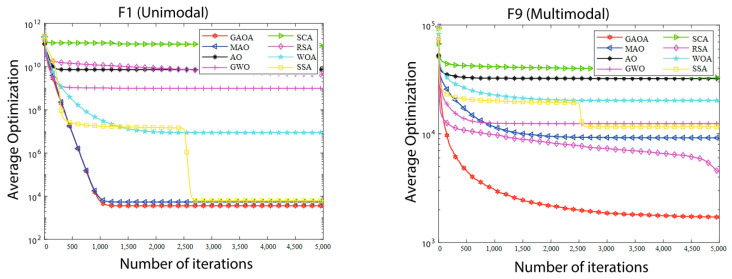

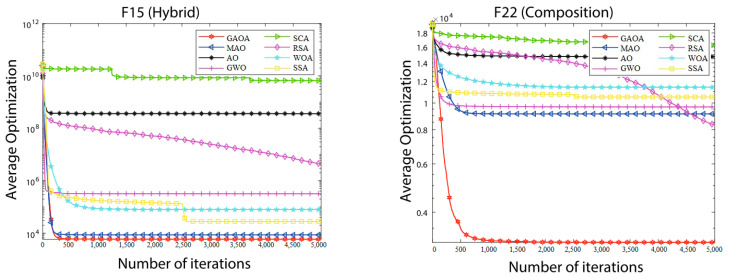
Convergence graphs of average optimizations obtained by 8 algorithms from CEC2017 functions (D = 50).

**Figure 4:**
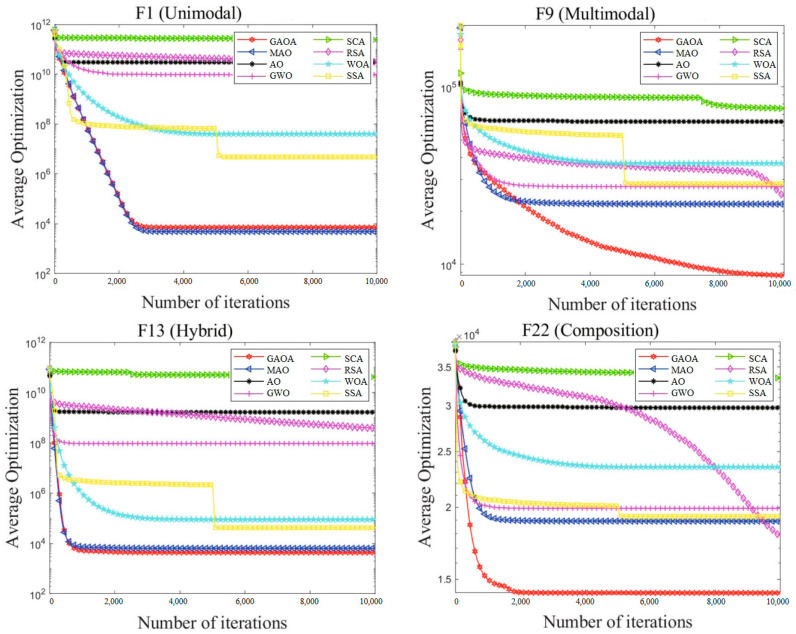
Convergence graphs of average optimizations obtained by 8 algorithms from CEC2017 functions (D = 100).

The convergence graphs of the average optimizations produced by eight algorithms on the IEEE CEC2017 functions with 30, 50, and 100 dimensions are shown in Figure 2, Figure 3 and Figure 4. The log value of the average optimizations is represented by the vertical axis, and the log value of iterations is represented by the horizontal axis. It is evident from Figure 2, Figure 3 and Figure 4 that the GAOA curves are the lowest and that the convergence speed is quick. The GAOA can identify a better solution, exit local optimization, prevent premature convergence, enhance the quality of the solution, and has a high optimization efficiency when compared to the original AO in the convergence graphs. This unequivocally proves the efficacy of the revised methodology presented in this research and the improvement in the population diversity. Unimodal functions do not entirely reflect the benefits of the GAOA. The GAOA may search for smaller values and converge quickly on increasingly complicated multimodal, hybrid, and composition functions, demonstrating excellent competitiveness.

### 4.3. Complexity of the Algorithm

The proposed algorithm’s usability and functionality are confirmed by the algorithm’s complexity. Due to their high computing cost, algorithms with high computational complexity are rarely investigated. Thus, for an algorithm to be effective, it must have strong optimization capabilities quick convergence and a minimal computational cost. In this section, we present the CPU running time used by all algorithms that were evaluated using IEEE CEC2017 functions with 30, 50, and 100 dimensions. We also address how the enhanced technique presented in this research affects the algorithm complexity of AO.

For each algorithm, the maximum number of function evaluations is fixed to be the same. Table 9 displays the findings for the CPU running time. The table shows that the WOA takes the least amount of time to compute. The GAOA and MAO require extremely little time to compute, and they have similar processing times. On the other hand, the RSA is the most difficult and time-consuming algorithm.

In Table 10, Table 11 and Table 12 Wilcoxon rank sum test is performed for the Table 3, Table 4 and Table 5 respectively to compare the *p*-values. And results shows that GAOA performs really good.

**Figure 5:**
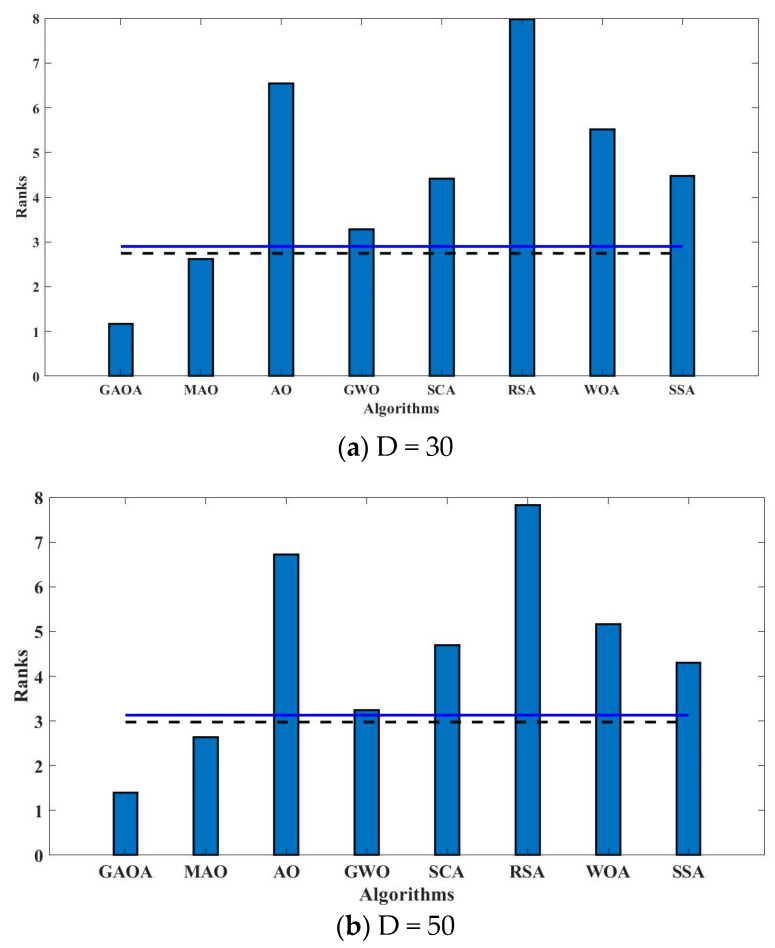

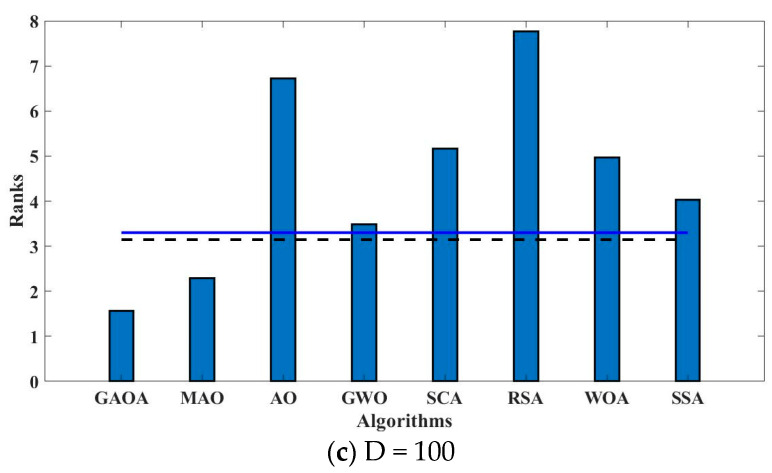
Bonferroni–Dunn bar chart for (**a**) D = 30, (**b**) D = 50, and (**c**) D = 100. The bar represents the rank of the correspondence algorithm, and horizontal cut lines show the significance level. (Here, ---- shows the significance level at 0.1, and 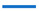 shows the significance level at 0.05.).

The metaheuristic algorithm performance is compared using the Bonferroni–Dunn bar chart. It is a trustworthy and dependable test that may be used to determine which algorithm performs the best for a specific set of benchmark functions. It is evident from Figure 5 that the GAOA outperforms the other metaheuristic methods. The eight algorithms are represented by the horizontal axis, while the rank is represented by the vertical axis. Future high-dimensional problems and engineering problems can benefit from the GAOA’s low computational complexity and low computational cost.

## 5. GAOA for Engineering Design Problems

The performance of the GAOA on the following five design problems is presented in this section to assess its effectiveness: pressure vessels, tension springs, three-bar trusses, speed reducers, and cantilever beams. A population size of 30 individuals and a maximum iteration of 500 were used to solve these problems. The results of the GAOA were then contrasted with other state-of-the-art algorithms that have been reported in the literature. The parameter settings used in the evaluation are consistent with those used in prior computational experiments.

### 5.1. Pressure Vessel Design Problem

**Figure 6:**
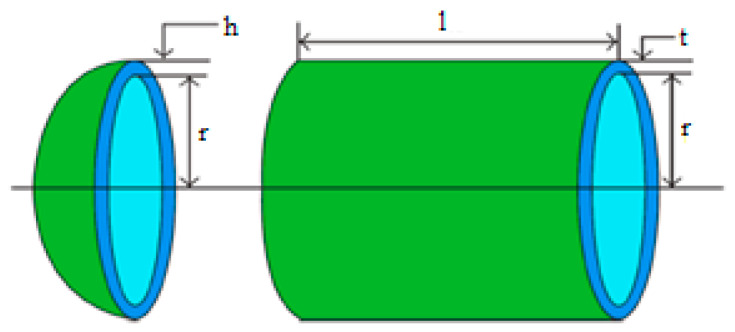
Pressure vessel design problem.

The pressure vessel design challenge [29,30] seeks to reduce the overall expense of a cylindrical pressure vessel while meeting the desired form and pressure criteria depicted in Figure 6. As shown in Figure 6, the answer to this problem entails minimizing four parameters: the shell’s thickness (t), head (h), cylindrical section sinner radius (r), and length without the top (l). The following are the issue’s restrictions and a corresponding equation.

Consider

$$s=[s_{1}\;s_{2}\;s_{3}\;s_{4}]=\left[t\;h\;r\;l\right]$$

Minimize

$$f(s)=0.6224s_{1}s_{3}s_{4}+1.7781s_{2}s_{3}^{2}+3.1661s_{1}^{2}s_{4}+19.84s_{1}^{2}s_{3}$$

Subject to

$$\begin{array}{c} g_{1}(s)=-s_{1}+0.0193s_{3}\leq 0,\\ g_{2}(s)=-s_{3}+0.00954s_{3}\leq 0, \end{array}$$

$$g_{3}(s)=-\pi s_{3}^{2}s_{4}-\frac{4}{3}\pi s_{3}^{3}+1,296,000\leq 0,$$

$$g_{4}(s)=s_{4}-240\leq 0.$$

The variable range is

$$\{\begin{array}{c} 0\leq s_{1}\leq 99,\\ 0\leq s_{2}\leq 99,\\ 10\leq s_{3}\leq 200,\\ 10\leq s_{4}\leq 200. \end{array}$$

Table 13’s results show that when the GAOA is compared to the COA, AO, GWO, ROA, RSA, WOA, and SCA, the ROA can obtain better ideal values.

### 5.2. Tension Spring Design Problem

**Figure 7:**
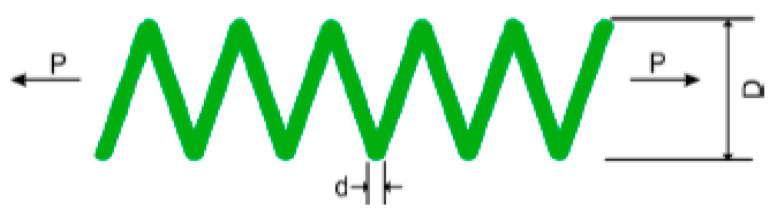
Tension spring design problem.

The three variables that needed to be tuned in order to optimize the design were the number of active coils (N), the mean coil diameter (D), and the wire diameter (d) [29,30]. Figure 7 shows the structural layout of the tension spring. The following is a presentation of the mathematical solution to this problem.

Consider

$$s=\left[s_{1}\;s_{2}\;s_{3}\;s_{4}\right]=\left[d\;D\;N\right]$$

Minimize

$$f(s)=(s_{3}+2)s_{2}s_{1}^{2}$$

Subject to

$$g_{1}(s)=1-\frac{s_{2}^{3}s_{3}}{71,785s_{1}^{4}}\leq 0,$$

$$g_{2}(s)=\frac{\left(4s_{2}^{2}-s_{1}s_{2}\right)}{12,566(s_{2}s_{1}^{3}-s_{1}^{4})}+\frac{1}{5108s_{1}^{2}}\leq 0,$$

$$g_{3}(s)=1-\frac{\left(140.45s_{1}\right)}{\left(s_{2}^{2}s_{3}\right)}\leq 0,$$

$$g_{4}\left(s\right)=\frac{s_{1}+s_{2}}{1.5}-1\leq 0$$

The variable range is

$$\begin{array}{l} 0.05\leq s_{1}\leq 2.00,\\ 0.25\leq s_{2}\leq 1.30,\\ 2.00\leq s_{3}\leq 15.00. \end{array}$$

Table 14 displays the outcomes of applying the GAOA to the tension spring design problem. The outcomes are then contrasted with those attained by a variety of other techniques, such as the COA, AO, GSA, DE, RSA, SMA, and EROA. It is evident that the COA produced outcomes that were superior to those of the other algorithms.

### 5.3. Three-Bar Truss Design Problem

**Figure 8:**
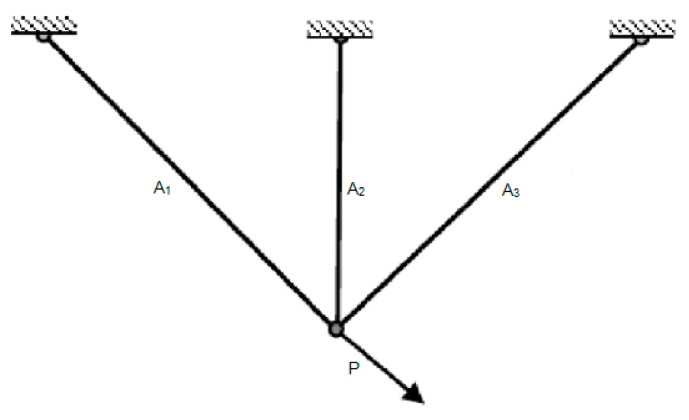
Three-bar truss design problem.

Designing a three-bar truss is a difficult problem in structural engineering [29,30]. The motto of this problem is to find a truss design that minimizes the weight while meeting the design constraint. Figure 8 shows the structural layout of the three-bar truss. The following is a presentation of the mathematical solution to this problem.

Consider

$$s=\left[s_{1}\;s_{2}\right]=\left[A_{1}\;A_{2}\right]$$

Minimize

$$f(s)=(2\sqrt{2}s_{1}+s_{2})*l$$

Subject to

$$g_{1}(s)=\frac{\left(\sqrt{2}s_{1}+s_{2}\right)}{\left(\sqrt{2}s_{1}^{2}+2s_{1}s_{2}\right)}P-\sigma \leq 0,$$

$$g_{2}(s)=\frac{s_{2}}{\left(\sqrt{2}s_{1}^{2}+2s_{1}s_{2}\right)}P-\sigma \leq 0,$$

$$g_{3}\left(s\right)=\frac{1}{\sqrt{2}s_{2}+s_{1}}P-\sigma \leq 0.$$

The variable range is

$$0\leq s_{1}$$

$$s_{2}\leq 1.$$

where l=100 cm, $P=2{\;\mathrm{KN}/\mathrm{cm}}^{2}$, and $\sigma =2{\;\mathrm{KN}/\mathrm{cm}}^{2}$.

The results of using the GAOA to solve the three-bar truss design problem are shown in Table 15. The results of these tests are then compared to those from the COA, GOA, AO, GWO, SSA, RSA, WOA, and SCA. It is clear that the GAOA has an exceptional ability to solve problems in a constrained environment.

### 5.4. Speed Reducer Problem

**Figure 9:**
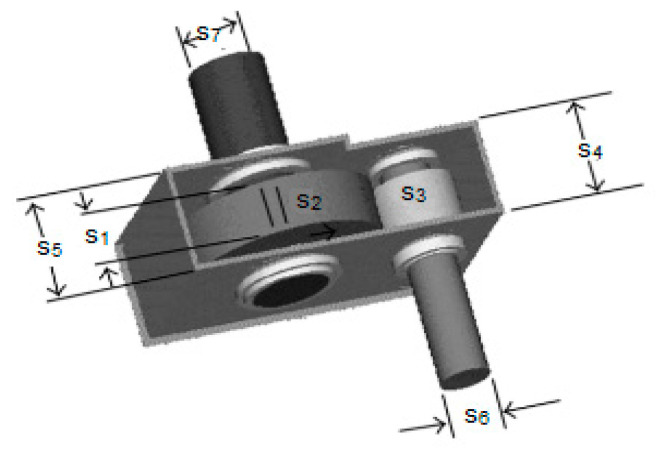
Speed reducer design problem.

By minimizing seven variables, the overall weight of the reducer in this problem [68] is reduced. The issue is laid out in Figure 9, and the solution is represented mathematically as follows:

Minimize

$$f(s)=0.7854s_{1}s_{2}^{2}(3.3333s_{3}^{2}+14.9334s_{3}-43.0934)-1.508s_{1}(s_{6}^{2}+s_{7}^{2})+7.4777(s_{6}^{3}+s_{7}^{3}),$$

Subject to

$$\begin{array}{l} g_{1}(s)=\frac{27}{s_{1}s_{2}^{2}s_{3}}-1\leq 0,\\ g_{2}(s)=\frac{397.5}{s_{1}s_{2}^{2}s_{3}^{2}}-1\leq 0, \end{array}$$

$$\begin{array}{l} g_{3}(s)=\frac{1.93s_{4}^{3}}{s_{2}s_{3}^{2}s_{6}^{4}}-1\leq 0,\\ g_{4}(s)=\frac{1.93s_{5}^{3}}{s_{2}s_{3}^{2}s_{7}^{4}}-1\leq 0, \end{array}$$

$$g_{5}(s)=\frac{\sqrt{\left({\left(\frac{745s_{4}}{s_{2}s_{3}}\right)}^{2}+16.9\times 10^{6}\right)}}{110.0s_{6}^{3}}-1\leq 0,$$

$$g_{6}(s)=\frac{\sqrt{\left({\left(\frac{745s_{4}}{s_{2}s_{3}}\right)}^{2}+157.5\times 10^{6}\right)}}{85.0s_{6}^{3}}-1\leq 0,$$

$$\begin{array}{l} g_{7}\left(s\right)=\frac{s_{2}s_{3}}{40}-1\leq 0,\\ g_{8}\left(s\right)=\frac{5s_{2}}{s_{1}}-1\leq 0, \end{array}$$

$$g_{9}(s)=\frac{s_{1}}{12s_{2}}-1\leq 0,$$

$$g_{10}(s)=\frac{1.5s_{6}+1.9}{s_{4}}-1\leq 0,$$

$$g_{11}(s)=\frac{1.1s_{7}+1.9}{s_{5}}-1\leq 0.$$

The variable range is

$$\begin{array}{c} 2.6\leq s_{1}\leq 3.6,\\ 0.7\leq s_{2}\leq 0.8, \end{array}$$

$$17\leq s_{3}\leq 28,$$

$$\begin{array}{c} 7.3\leq s_{4}\leq 8.3,\\ 7.8\leq s_{5}\leq 8.3,\\ 2.9\leq s_{6}\leq 3.9,\\ 5.0\leq s_{7}\leq 5.5. \end{array}$$

Table 16 displays the comparison findings and the benefit of using the GAOA to achieve the smallest overall weight of the problem.

### 5.5. Cantilever Beam Design

The determination of the least overall weight of cantilever beams is a specific problem in concrete engineering. The thickness of the walls of the hollow square cross section, as well as the dimensions of the square, can all affect the weight.

**Figure 10:**
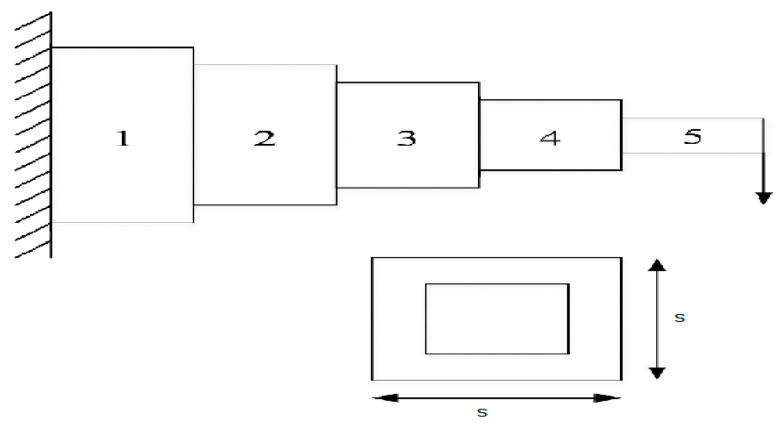
Cantilever beam design problem.

The objective of the optimization problem is to find the values of these parameters that minimize the overall weight of the beam while still ensuring that the beam is strong enough to withstand the applied load [29]. The design configuration associated with this problem is depicted visually in Figure 10, and it can be represented mathematically using the following formulation:

Consider

$$s=\left[s_{1}\;s_{2}\;s_{3}\;s_{4}\;s_{5}\right]$$

Minimize

$$f(s)=0.6224(s_{1}+s_{2}+s_{3}+s_{4}+s_{5}),$$

Subject to

$$g(s)=\frac{60}{s_{1}^{3}}+\frac{27}{s_{2}^{3}}+\frac{19}{s_{3}^{3}}+\frac{7}{s_{4}^{3}}+\frac{1}{s_{5}^{3}}-1\leq 0$$

The variable range is

$$0.01\leq s_{1},s_{2},s_{3},s_{4},s_{5}\leq 100.$$

The results in Table 17 show that the GAOA achieves the minimized overall weight faster and with a better performance than all other algorithms. In conclusion, this section emphasizes how excellent the suggested GAOA is in comparison to other characteristics and real-world case studies. With extremely competitive results, the GAOA displays its capacity to outperform both the fundamental COA and ROA algorithms as well as other well-known algorithms. These successes are a result of the GAOA’s strong exploration and exploitation capabilities. Its outstanding success in resolving industrial engineering design issues further highlights its potential for widespread use in practical optimization issues.

## 6. Conclusions

- This article introduces the GAOA, which is the modification of the entire Aquila Optimizer (AO). To improve the GAOA’s capacities for exploration and exploitation, a mutation opposition-based learning strategy is used.
- Then, 23 classical benchmark functions and the CEC 2017 benchmark test functions are used to assess the GAOA’s performance and examine its exploration capability, exploitation capability, and ability to avoid stagnation. The experimental results highlight the GAOA’s better performance and competitive benefits compared to other cutting-edge metaheuristic algorithms.
- Five engineering design challenges are successfully solved using the algorithm, further demonstrating its superiority to previous metaheuristic algorithms. The suggested GAOA handles complex benchmark functions and limited engineering issues with surprising effectiveness.
- The GAOA has the potential to be used in the future for a variety of practical optimization issues, such as problems in multi-objective, feature selection, multi-threshold image segmentation, convolutional neural networks, and NP-hard issues.

## 7. Future Scope

The GAOA could be applied in additional real-world applications given its great performance. Additionally, other optimization jobs, including image processing, cloud and fog computing, and others, could use the GAOA optimization method.

## Figures and Tables

**Table 1:** Classification of algorithms.

Evolution-Inspired	Genetic Algorithm (GA) [24] Differential Evolution (DE) [25] Bat Algorithm (BA) [26] Bacterial Foraging Optimization (BFO) [27] Artificial Immune System (AIS) [28]
Swarm-Inspired	Aquila Optimizer (AO) [29] Grey Wolf Optimizer (GWO) [30] Whale Optimization Algorithm (WOA) [31] Reptile Search Algorithm (RSA) [32] Particle Swarm Optimization (PSO) [33] Salp Swarm Algorithm (SSA) [34] Sine Cosine Algorithm (SCA) [35] Dynamic Harris Hawks Optimization (DHHO) [36]
Physics-Inspired	Gravitational Search Algorithm (GSA) [37] Wind Driven Optimization (WDO) [38] Atom Search Optimization (ASO) [39]
Human-Inspired	Teaching–Learning-Based Optimization (TLBO) [40] Poor and Rich Optimization (PRO) [41]

**Table 2:** Parameter settings for GAOA and other algorithms.

Algorithm	Parameters
GAOA	$a:2\to 0,\;U=0.00565,\;r=10,\;\omega =0.05,\;\alpha =0.1,\;\delta =0.1,P_{1}\in \left[-1,1\right],P_{2}=\left[2,0\right]$
MAO	$U=0.00565,\;r=10,\;\omega =0.05,\;\alpha =0.1,\;\delta =0.1,P_{1}\in \left[-1,1\right],P_{2}=\left[2,0\right]$
AO	$U=0.00565,\;r=10,\;\omega =0.05,\;\alpha =0.1,\;\delta =0.1,P_{1}\in \left[-1,1\right],P_{2}=\left[2,0\right]$
GWO	a:2→0
SCA	a=[2,0]
RSA	α=0.1,β=0.005
WOA	$w_{1}=\left[2,0\right],w_{2}=\left[-1,-2\right],v=1$
SSA	$s_{1}=\left[1,0\right],s_{2}\in \left[0.1\right],s_{3}\in \left[0,1\right]$

**Table 3:** GAOA and seven competing algorithms’ experimental and statistical data on the benchmark functions with 30 dimensions from IEEE CEC2017.

Function	GAOA	MAO	AO	GWO	SCA	RSA	WOA	SSA
F1 Mean STD	2.512 × 10^3^ 3.401 × 10^3^	3.641 × 10^3^ 4.381 × 10^3^	1.511 × 10^9^ 7.720 × 10^9^	9.328 × 10^8^ 8.157 × 10^8^	1.661 × 10^7^ 8.017 × 10^7^	5.384 × 10 9.292 × 10^9^	2.826 × 10^7^ 1.911 × 10^7^	2.410 × 10^3^ 2.501 × 10^3^
F3 Mean STD	5.501 × 10^1^ 4.013 × 10^1^	4.823 × 10^0^ 1.101 × 10^1^	5.731 × 10^4^ 3.812 × 10^4^	2.803 × 10^4^ 8.092 × 10^3^	2.429 × 10^3^ 4.748 × 10^3^	7.423 × 10^4^ 5.505 × 10^4^	1.642 × 10^4^ 6.126 × 10^5^	3.001 × 10^1^ 1.710 × 10^2^
F4 Mean STD	3.103 × 10^1^ 3.571 × 10^1^	4.579 × 10^1^ 3.631 × 10^1^	6.081 × 10^2^ 1.433 × 10^3^	1.442 × 10^2^ 3.276 × 10^1^	4.581 × 10^2^ 3.170 × 10^2^	1.455 × 10^4^ 4.561 × 10^3^	1.588 × 10^2^ 4.127 × 10^1^	9.495 × 10^1^ 2.001 × 10^1^
F5 Mean STD	6.071 × 10^1^ 1.581 × 10^1^	1.642 × 10^2^ 4.233 × 10^1^	2.911 × 10^2^ 8.777 × 10^1^	9.191 × 10^1^ 2.354 × 10^1^	1.457 × 10^2^ 4.729 × 10^1^	3.891 × 10^2^ 3.309 × 10^1^	2.743 × 10^2^ 5.211 × 10^1^	2.038 × 10^2^ 4.101 × 10^1^
F6 Mean STD	2.091 × 10^0^ 3.030 × 10^−1^	1.871 × 10^1^ 1.102 × 10^1^	6.241 × 10^1^ 1.873 × 10^1^	4.693 × 10^0^ 3.004 × 10^0^	2.502 × 10^1^ 8.602 × 10^0^	8.634 × 10^1^ 7.461 × 10^0^	6.627 × 10^1^ 9.812 × 10^0^	5.316 × 10^1^ 6.414 × 10^0^
F7 Mean STD	1.100 × 10^2^ 2.710 × 10^1^	2.611 × 10^2^ 7.001 × 10^1^	4.799 × 10^2^ 1.533 × 10^2^	1.474 × 10^2^ 3.596 × 10^1^	2.569 × 10^2^ 5.750 × 10^1^	6.725 × 10^2^ 6.730 × 10^1^	5.470 × 10^2^ 9.151 × 10^1^	5.110 × 10^2^ 1.011 × 10^2^
F8 Mean STD	6.812 × 10^1^ 2.251 × 10^1^	1.190 × 10^2^ 3.460 × 10^1^	2.122 × 10^2^ 8.900 × 10^1^	8.182 × 10^1^ 2.299 × 10^1^	1.273 × 10^2^ 2.980 × 10^1^	3.116 × 10^2^ 2.206 × 10^1^	2.031 × 10^2^ 5.167 × 10^1^	1.451 × 10^2^ 3.211 × 10^1^
F9 Mean STD	4.485 × 10^1^ 4.790 × 10^1^	2.304 × 10^3^ 1.152 × 10^3^	7.801 × 10^3^ 3.310 × 10^3^	4.242 × 10^2^ 2.347 × 10^2^	3.154 × 10^3^ 1.325 × 10^3^	8.533 × 10^3^ 1.196 × 10^3^	6.089 × 10^3^ 2.090 × 10^3^	3.411 × 10^3^ 6.754 × 10^2^
F10 Mean STD	3.430 × 10^3^ 5.75 × 10^2^	3.660 × 10^3^ 6.391 × 10^2^	6.411 × 10^3^ 1.734 × 10^3^	2.909 × 10^3^ 7.536 × 10^2^	3.524 × 10^3^ 7.312 × 10^2^	7.021 × 10^3^ 3.593 × 10^2^	5.121 × 10^3^ 8.401 × 10^2^	4.211 × 10^3^ 6.081 × 10^2^
F11 Mean STD	6.901 × 10^1^ 2.791 × 10^1^	8.771 × 10^1^ 3.543 × 10^1^	2.031 × 10^3^ 2.331 × 10^3^	3.375 × 10^2^ 3.685 × 10^2^	3.566 × 10^2^ 2.507 × 10^2^	7.770 × 10^3^ 2.806 × 10^3^	4.111 × 10^2^ 1.331 × 10^2^	1.378 × 10^2^ 4.511 × 10^1^
F12 Mean STD	4.531 × 10^4^ 2.510 × 10^4^	6.078 × 10^4^ 2.860 × 10^4^	3.713 × 10^8^ 9.301 × 10^8^	3.088 × 10^7^ 5.277 × 10^7^	8.585 × 10^7^ 1.659 × 10^8^	1.703 × 10^10^ 4.663 × 10^9^	3.911 × 10^7^ 3.199 × 10^7^	1.614 × 10^6^ 8.094 × 10^5^
F13 Mean STD	7.900 × 10^3^ 8.512 × 10^3^	1.311 × 10^4^ 1.431 × 10^4^	4.823 × 10^8^ 1.011 × 10^9^	6.233 × 10^5^ 3.575 × 10^6^	1.201 × 10^7^ 4.615 × 10^7^	1.187 × 10^10^ 4.906 × 10^9^	1.297 × 10^5^ 1.194 × 10^5^	5.216 × 10^4^ 2.340 × 10^4^
F14 Mean STD	1.463 × 10^3^ 1.501 × 10^3^	2.810 × 10^3^ 3.311 × 10^3^	6.001 × 10^5^ 6.399 × 10^5^	1.484 × 10^5^ 2.439 × 10^5^	1.356 × 10^5^ 2.768 × 10^5^	3.074 × 10^6^ 3.588 × 10^6^	7.001 × 10^5^ 6.901 × 10^5^	4.170 × 10^3^ 3.152 × 10^3^
F15 Mean STD	2.911 × 10^3^ 3.221 × 10^3^	7.241 × 10^3^ 9.211 × 10^3^	2.412 × 10^7^ 5.101 × 10^7^	9.164 × 10^4^ 2.899 × 10^5^	2.104 × 10^5^ 1.366 × 10^6^	6.736 × 10^8^ 5.747 × 10^8^	7.581 × 10^4^ 5.291 × 10^4^	3.112 × 10^4^ 2.122 × 10^4^
F16 Mean STD	7.605 × 10^2^ 2.751 × 10^2^	9.101 × 10^2^ 2.300 × 10^2^	2.092 × 10^3^ 8.831 × 10^2^	7.493 × 10^2^ 2.635 × 10^2^	1.328 × 10^3^ 3.729 × 10^2^	3.898 × 10^3^ 6.862 × 10^2^	2.005 × 10^3^ 4.610 × 10^2^	1.504 × 10^3^ 3.314 × 10^2^
F17 Mean STD	1.742 × 10^2^ 1.100 × 10^2^	4.563 × 10^2^ 2.204 × 10^2^	9.123 × 10^2^ 4.001 × 10^2^	2.779 × 10^2^ 1.651 × 10^2^	5.103 × 10^2^ 2.027 × 10^2^	5.306 × 10^3^ 6.866 × 10^3^	7.780 × 10^2^ 2.780 × 10^2^	8.120 × 10^2^ 2.401 × 10^2^
F18 Mean STD	8.014 × 10^4^ 4.841 × 10^4^	8.612 × 10^4^ 3.800 × 10^4^	6.833 × 10^6^ 9.212 × 10^6^	6.210 × 10^5^ 5.727 × 10^5^	6.259 × 10^5^ 2.004 × 10^6^	3.277 × 10^7^ 3.071 × 10^7^	2.940 × 10^6^ 2.656 × 10^6^	1.120 × 10^5^ 1.001 × 10^5^
F19 Mean STD	5.611 × 10^3^ 6.400 × 10^3^	8.194 × 10^3^ 9.885 × 10^3^	3.514 × 10^7^ 7.989 × 10^7^	8.827 × 10^5^ 1.949 × 10^6^	1.493 × 10^4^ 1.536 × 10^4^	2.323 × 10^9^ 1.694 × 10^9^	2.747 × 10^6^ 2.061 × 10^6^	1.301 × 10^5^ 5.361 × 10^4^
F20 Mean STD	2.100 × 10^2^ 8.214 × 10^1^	4.501 × 10^2^ 1.824 × 10^2^	7.711 × 10^2^ 1.981 × 10^2^	3.404 × 10^2^ 1.337 × 10^2^	6.274 × 10^2^ 2.118 × 10^2^	8.636 × 10^2^ 1.426 × 10^2^	7.130 × 10^2^ 2.025 × 10^2^	7.219 × 10^2^ 2.015 × 10^2^
F21 Mean STD	2.462 × 10^2^ 1.610 × 10^1^	3.101 × 10^2^ 3.341 × 10^1^	4.612 × 10^2^ 1.154 × 10^2^	2.834 × 10^2^ 2.986 × 10^1^	3.351 × 10^2^ 4.075 × 10^1^	6.431 × 10^2^ 4.269 × 10^1^	4.510 × 10^2^ 5.431 × 10^1^	4.004 × 10^2^ 4.051 × 10^1^
F22 Mean STD	1.010 × 10^2^ 6.702 × 10^−1^	1.114 × 10^2^ 1.113 × 10^0^	5.511 × 10^2^ 1.304 × 10^3^	1.762 × 10^3^ 1.485 × 10^3^	2.591 × 10^3^ 2.065 × 10^3^	5.253 × 10^3^ 1.008 × 10^3^	4.401 × 10^3^ 2.103 × 10^3^	4.001 × 10^3^ 1.701 × 10^3^
F23 Mean STD	4.214 × 10^2^ 2.750 × 10^1^	4.923 × 10^2^ 3.701 × 10^1^	8.330 × 10^2^ 1.502 × 10^2^	4.321 × 10^2^ 2.277 × 10^1^	6.291 × 10^2^ 1.227 × 10^2^	1.039 × 10^3^ 1.089 × 10^2^	7.206 × 10^2^ 9.760 × 10^1^	9.991 × 10^2^ 1.013 × 10^2^
F24 Mean STD	5.016 × 10^2^ 3.011 × 10^1^	5.712 × 10^2^ 5.281 × 10^1^	8.700 × 10^2^ 1.711 × 10^2^	5.032 × 10^2^ 4.536 × 10^1^	7.538 × 10^2^ 1.073 × 10^2^	1.177 × 10^3^ 2.453 × 10^2^	7.810 × 10^2^ 8.921 × 10^1^	1.004 × 10^3^ 9.711 × 10^1^
F25 Mean STD	3.212 × 10^2^ 2.310 × 10^0^	4.011 × 10^2^ 1.910 × 10^1^	6.799 × 10^2^ 5.582 × 10^2^	4.570 × 10^2^ 2.613 × 10^1^	5.397 × 10^2^ 1.008 × 10^2^	2.224 × 10^3^ 8.605 × 10^2^	4.500 × 10^2^ 3.001 × 10^1^	4.101 × 10^2^ 9.110 × 10^0^
F26 Mean STD	1.302 × 10^3^ 9.812 × 10^2^	2.041 × 10^3^ 1.511 × 10^3^	3.341 × 10^3^ 2.261 × 10^3^	1.837 × 10^3^ 3.100 × 10^2^	3.121 × 10^3^ 1.143 × 10^3^	7.933 × 10^3^ 1.124 × 10^3^	4.912 × 10^3^ 9.810 × 10^2^	5.832 × 10^3^ 1.112 × 10^3^
F27 Mean STD	5.322 × 10^2^ 1.876 × 10^1^	5.711 × 10^2^ 2.821 × 10^1^	9.114 × 10^2^ 1.990 × 10^2^	5.326 × 10^2^ 1.520 × 10^1^	8.132 × 10^2^ 9.760 × 10^1^	9.409 × 10^2^ 2.313 × 10^2^	6.499 × 10^2^ 6.500 × 10^1^	1.204 × 10^3^ 2.510 × 10^2^
F28 Mean STD	3.120 × 10^2^ 4.670 × 10^1^	3.406 × 10^2^ 5.676 × 10^1^	7.651 × 10^2^ 8.041 × 10^2^	5.472 × 10^2^ 5.758 × 10^1^	7.495 × 10^2^ 2.564 × 10^2^	3.985 × 10^3^ 8.850 × 10^2^	5.001 × 10^2^ 3.121 × 10^1^	3.854 × 10^2^ 5.120 × 10^1^
F29 Mean STD	6.821 × 10^2^ 1.512 × 10^2^	9.094 × 10^2^ 2.071 × 10^2^	2.041 × 10^3^ 6.110 × 10^2^	7.531 × 10^2^ 1.339 × 10^2^	1.322 × 10^3^ 4.000 × 10^2^	4.146 × 10^3^ 1.609 × 10^3^	2.001 × 10^3^ 4.100 × 10^2^	1.520 × 10^3^ 3.701 × 10^2^
F30 Mean STD	4.811 × 10^3^ 2.123 × 10^3^	6.001 × 10^3^ 2.612 × 10^3^	5.601 × 10^7^ 8.344 × 10^7^	5.504 × 10^6^ 5.643 × 10^6^	1.681 × 10^6^ 4.255 × 10^6^	2.239 × 10^9^ 9.259 × 10^8^	9.787 × 10^6^ 6.828 × 10^6^	5.371 × 10^5^ 3.104 × 10^5^
**(W/L/T)** **Average Rank**	-/-/- 1.17	28/1/0 2.62	29/0/0 6.55	27/2/0 3.28	29/0/0 4.41	29/0/0 7.97	29/0/0 5.52	27/2/0 4.48

**Table 4:** GAOA and seven competing algorithms’ experimental and statistical data on the benchmark functions with 50 dimensions from IEEE CEC2017.

Function	GAOA	MAO	AO	GWO	SCA	RSA	WOA	SSA
F1 Mean STD	3.667 × 10^3^ 4.401 × 10^3^	5.365 × 10^3^ 5.327 × 10^3^	7.360 × 10^9^ 2.171 × 10^10^	4.493 × 10^9^ 2.326 × 10^9^	9.919 × 10^8^ 3.411 × 10^9^	9.635 × 10^10^ 9.884 × 10^9^	8.668 × 10^6^ 8.653 × 10^6^	6.232 × 10^3^ 5.212 × 10^3^
F3 Mean STD	2.912 × 10^4^ 5.334 × 10^3^	1.624 × 10^4^ 4.453 × 10^3^	2.041 × 10^5^ 4.305 × 10^4^	7.261 × 10^4^ 1.523 × 10^4^	4.166 × 10^4^ 5.777 × 10^4^	1.487 × 10^5^ 1.042 × 10^4^	6.664 × 10^4^ 3.301 × 10^4^	5.412 × 10^2^ 7.890 × 10^2^
F4 Mean STD	9.332 × 10^1^ 5.876 × 10^1^	9.010 × 10^1^ 5.124 × 10^1^	1.528 × 10^3^ 4.977 × 10^3^	4.341 × 10^2^ 1.771 × 10^2^	3.289 × 10^3^ 1.935 × 10^3^	2.710 × 10^4^ 6.760 × 10^3^	3.101 × 10^2^ 7.122 × 10^1^	1.711 × 10^2^ 4.724 × 10^1^
F5 Mean STD	1.621 × 10^2^ 3.990 × 10^1^	3.304 × 10^2^ 3.742 × 10^1^	4.251 × 10^2^ 1.586 × 10^2^	1.922 × 10^2^ 5.205 × 10^1^	3.091 × 10^2^ 6.603 × 10^1^	6.336 × 10^2^ 2.898 × 10^1^	4.235 × 10^2^ 8.622 × 10^1^	3.266 × 10^2^ 4.253 × 10^1^
F6 Mean STD	4.359 × 10^0^ 4.566 × 10^0^	3.900 × 10^1^ 1.127 × 10^1^	8.072 × 10^1^ 1.928 × 10^1^	1.019 × 10^1^ 3.623 × 10^0^	3.588 × 10^1^ 8.211 × 10^0^	9.827 × 10^1^ 4.680 × 10^0^	7.823 × 10^1^ 1.124 × 10^1^	6.011 × 10^1^ 4.134 × 10^0^
F7 Mean STD	2.481 × 10^2^ 5.343 × 10^1^	6.432 × 10^2^ 1.432 × 10^2^	9.171 × 10^2^ 1.878 × 10^2^	3.037 × 10^2^ 6.764 × 10^1^	6.691 × 10^2^ 1.175 × 10^2^	1.302 × 10^3^ 5.902 × 10^1^	1.110 × 10^3^ 9.324 × 10^1^	1.142 × 10^3^ 1.421 × 10^2^
F8 Mean STD	1.678 × 10^2^ 4.734 × 10^1^	3.352 × 10^2^ 5.221 × 10^1^	5.012 × 10^2^ 1.882 × 10^2^	1.852 × 10^2^ 3.069 × 10^1^	3.035 × 10^2^ 6.896 × 10^1^	6.791 × 10^2^ 2.650 × 10^1^	4.424 × 10^2^ 9.601 × 10^1^	3.422 × 10^2^ 4.754 × 10^1^
F9 Mean STD	8.113 × 10^2^ 6.756 × 10^2^	8.370 × 10^3^ 2.628 × 10^3^	3.127 × 10^4^ 1.240 × 10^4^	3.551 × 10^3^ 2.143 × 10^3^	1.151 × 10^4^ 3.209 × 10^3^	3.204 × 10^4^ 2.580 × 10^3^	2.012 × 10^4^ 4.745 × 10^3^	1.143 × 10^4^ 1.324 × 10^3^
F10 Mean STD	6.212 × 10^3^ 1.012 × 10^3^	6.646 × 10^3^ 8.435 × 10^2^	1.173 × 10^4^ 3.014 × 10^3^	3.551 × 10^3^ 2.143 × 10^3^	5.973 × 10^3^ 1.019 × 10^3^	1.315 × 10^4^ 4.800 × 10^2^	8.650 × 10^3^ 1.342 × 10^3^	7.251 × 10^3^ 8.125 × 10^2^
F11 Mean STD	1.365 × 10^2^ 3.434 × 10^1^	1.657 × 10^2^ 3.951 × 10^1^	6.883 × 10^3^ 8.539 × 10^3^	1.789 × 10^3^ 1.152 × 10^3^	4.105 × 10^3^ 4.079 × 10^3^	1.682 × 10^4^ 2.846 × 10^3^	5.012 × 10^2^ 1.126 × 10^2^	2.131 × 10^2^ 4.236 × 10^1^
F12 Mean STD	5.465 × 10^5^ 3.254 × 10^5^	7.611 × 10^5^ 4.842 × 10^5^	4.109 × 10^9^ 1.312 × 10^10^	5.809 × 10^8^ 6.600 × 10^8^	1.296 × 10^9^ 2.252 × 10^9^	7.667 × 10^10^ 1.708 × 10^10^	2.112 × 10^8^ 1.145 × 10^8^	1.375 × 10^7^ 7.012 × 10^6^
F13 Mean STD	2.633 × 10^3^ 3.555 × 10^3^	4.113 × 10^3^ 5.001 × 10^3^	1.521 × 10^9^ 3.723 × 10^9^	1.168 × 10^8^ 1.135 × 10^8^	2.332 × 10^6^ 1.638 × 10^7^	4.590 × 10^10^ 1.270 × 10^10^	2.724 × 10^5^ 2.241 × 10^5^	6.153 × 10^4^ 3.245 × 10^4^
F14 Mean STD	2.234 × 10^4^ 1.454 × 10^4^	2.101 × 10^4^ 2.122 × 10^4^	6.639 × 10^6^ 7.101 × 10^6^	3.512 × 10^5^ 3.476 × 10^5^	4.044 × 10^6^ 6.734 × 10^6^	3.605 × 10^7^ 2.864 × 10^7^	6.745 × 10^5^ 4.243 × 10^5^	3.121 × 10^4^ 2.553 × 10^4^
F15 Mean STD	4.465 × 10^3^ 4.219 × 10^3^	7.128 × 10^3^ 6.524 × 10^3^	3.622 × 10^8^ 6.853 × 10^8^	4.375 × 10^6^ 9.979 × 10^6^	3.162 × 10^5^ 1.642 × 10^6^	6.628 × 10^9^ 4.800 × 10^9^	7.646 × 10^4^ 5.112 × 10^4^	2.745 × 10^4^ 1.210 × 10^4^
F16 Mean STD	1.267 × 10^3^ 4.343 × 10^2^	1.757 × 10^3^ 4.703 × 10^2^	3.691 × 10^3^ 1.479 × 10^3^	1.280 × 10^3^ 3.636 × 10^2^	2.631 × 10^3^ 7.796 × 10^2^	7.172 × 10^3^ 1.378 × 10^3^	3.123 × 10^3^ 6.645 × 10^2^	2.321 × 10^3^ 5.354 × 10^2^
F17 Mean STD	1.121 × 10^3^ 2.988 × 10^2^	1.251 × 10^3^ 3.423 × 10^2^	2.407 × 10^3^ 6.650 × 10^2^	9.308 × 10^2^ 2.273 × 10^2^	1.516 × 10^3^ 3.538 × 10^2^	1.819 × 10^4^ 3.409 × 10^4^	2.382 × 10^3^ 4.546 × 10^2^	2.011 × 10^3^ 4.024 × 10^2^
F18 Mean STD	2.121 × 10^5^ 1.233 × 10^5^	1.572 × 10^5^ 8.769 × 10^4^	2.837 × 10^7^ 3.638 × 10^7^	4.024 × 10^6^ 4.810 × 10^6^	1.048 × 10^7^ 1.422 × 10^7^	9.345 × 10^7^ 5.237 × 10^7^	5.124 × 10^6^ 4.163 × 10^6^	2.512 × 10^5^ 1.110 × 10^5^
F19 Mean STD	1.478 × 10^4^ 8.260 × 10^3^	1.545 × 10^4^ 1.231 × 10^4^	1.635 × 10^8^ 3.962 × 10^8^	1.429 × 10^6^ 2.421 × 10^6^	1.283 × 10^4^ 1.670 × 10^4^	6.888 × 10^9^ 2.802 × 10^9^	1.901 × 10^6^ 1.232 × 10^6^	4.882 × 10^5^ 2.534 × 10^5^
F20 Mean STD	8.364 × 10^2^ 2.834 × 10^2^	1.119 × 10^3^ 3.662 × 10^2^	1.797 × 10^3^ 4.564 × 10^2^	7.867 × 10^2^ 3.287 × 10^2^	1.230 × 10^3^ 3.499 × 10^2^	1.825 × 10^3^ 1.962 × 10^2^	1.721 × 10^3^ 3.121 × 102	1.421 × 10^3^ 3.012 × 10^2^
F21 Mean STD	3.387 × 10^2^ 2.322 × 10^1^	4.599 × 10^2^ 5.285 × 10^1^	6.837 × 10^2^ 1.790 × 10^2^	3.815 × 10^2^ 2.742 × 10^1^	5.105 × 10^2^ 8.051 × 10^1^	1.032 × 10^3^ 9.733 × 10^1^	7.631 × 10^2^ 1.172 × 10^2^	6.470 × 10^2^ 6.612 × 10^1^
F22 Mean STD	9.125 × 10^2^ 2.343 × 10^3^	7.036 × 10^3^ 1.692 × 10^3^	1.255 × 10^4^ 3.307 × 10^3^	6.136 × 10^3^ 1.341 × 10^3^	7.477 × 10^3^ 1.474 × 10^3^	1.408 × 10^4^ 5.151 × 10^2^	9.101 × 10^3^ 1.231 × 10^3^	8.325 × 10^3^ 8.670 × 10^2^
F23 Mean STD	6.235 × 10^2^ 5.421 × 10^1^	7.943 × 10^2^ 9.845 × 10^1^	1.463 × 10^3^ 2.659 × 10^2^	6.181 × 10^2^ 5.885 × 10^1^	1.078 × 10^3^ 2.207 × 10^2^	1.654 × 10^3^ 1.582 × 10^2^	1.342 × 10^3^ 1.244 × 10^2^	1.720 × 10^3^ 2.150 × 10^2^
F24 Mean STD	6.642 × 10^2^ 4.964 × 10^1^	8.741 × 10^2^ 9.613 × 10^1^	1.548 × 10^3^ 2.956 × 10^2^	7.142 × 10^2^ 9.997 × 10^1^	1.423 × 10^3^ 3.037 × 10^2^	2.022 × 10^3^ 4.130 × 10^2^	1.346 × 10^3^ 1.623 × 10^2^	1.732 × 10^3^ 2.121 × 10^2^
F25 Mean STD	5.625 × 10^2^ 3.253 × 10^1^	5.634 × 10^2^ 3.725 × 10^1^	1.465 × 10^3^ 2.862 × 10^3^	8.599 × 10^2^ 1.553 × 10^2^	2.082 × 10^3^ 7.709 × 10^2^	1.031 × 10^4^ 1.269 × 10^3^	6.512 × 10^2^ 3.453 × 10^1^	5.654 × 10^2^ 3.430 × 10^1^
F26 Mean STD	2.250 × 10^3^ 2.437 × 10^3^	3.888 × 10^3^ 3.801 × 10^3^	7.648 × 10^3^ 4.114 × 10^3^	3.201 × 10^3^ 5.942 × 10^2^	7.099 × 10^3^ 1.543 × 10^3^	1.341 × 10^4^ 1.036 × 10^3^	1.112 × 10^4^ 1.434 × 10^3^	1.151 × 10^4^ 8.041 × 10^2^
F27 Mean STD	7.865 × 10^2^ 9.353 × 10^1^	9.120 × 10^2^ 1.554 × 10^2^	7.648 × 10^3^ 4.114 × 10^3^	7.910 × 10^2^ 7.505 × 10^1^	2.078 × 10^3^ 3.871 × 10^2^	1.905 × 10^3^ 3.265 × 10^2^	1.232 × 10^3^ 3.432 × 10^2^	2.630 × 10^3^ 4.821 × 10^2^
F28 Mean STD	4.994 × 10^2^ 2.865 × 10^1^	5.011 × 10^2^ 3.343 × 10^1^	1.290 × 10^3^ 1.762 × 10^3^	1.032 × 10^3^ 2.321 × 10^2^	2.944 × 10^3^ 8.029 × 10^2^	8.950 × 10^3^ 1.133 × 10^3^	6.341 × 10^2^ 5.243 × 10^1^	5.036 × 10^2^ 3.097 × 10^1^
F29 Mean STD	1.122 × 10^3^ 3.287 × 10^2^	1.543 × 10^3^ 3.122 × 10^2^	4.766 × 10^3^ 3.884 × 10^3^	1.316 × 10^3^ 2.674 × 10^2^	3.052 × 10^3^ 8.637 × 10^2^	6.189 × 10^4^ 4.451 × 10^4^	4.240 × 10^3^ 9.363 × 10^2^	2.712 × 10^3^ 4.751 × 10^2^
F30 Mean STD	8.824 × 10^5^ 1.674 × 10^5^	9.655 × 10^5^ 2.112 × 10^5^	4.685 × 10^8^ 9.958 × 10^8^	7.309 × 10^7^ 3.310 × 10^7^	9.298 × 10^7^ 6.349 × 10^7^	8.605 × 10^9^ 2.718 × 10^9^	8.642 × 10^7^ 3.010 × 10^7^	1.491 × 10^7^ 1.961 × 10^6^
**(W/L/T)** **Average Rank**	-/-/- 1.40	21/3/4 2.64	29/0/1 6.72	25/4/0 3.24	29/0/0 4.69	29/0/0 7.83	29/0/0 5.17	28/1/0 4.31

**Table 5:** GAOA and seven competing algorithms’ experimental and statistical data on the benchmark functions with 100 dimensions from IEEE CEC2017.

Function	GAOA	MAO	AO	GWO	SCA	RSA	WOA	SSA
F1 Mean STD	7.001 × 10^3^ 1.120 × 10^4^	4.734 × 10^3^ 7.011 × 10^3^	3.011 × 10^10^ 8.314 × 10^10^	3.186 × 10^10^ 6.811 × 10^9^	9.611 × 10^9^ 2.720 × 10^10^	2.435 × 10^11^ 9.446 × 10^9^	4.001 × 10^7^ 1.812 × 10^7^	4.701 × 10^6^ 1.312 × 10^6^
F3 Mean STD	1.323 × 10^5^ 1.790 × 10^4^	8.843 × 10^4^ 1.441 × 10^4^	4.011 × 10^5^ 1.210 × 10^5^	2.030 × 10^5^ 2.311 × 10^4^	1.290 × 10^5^ 1.210 × 10^5^	3.155 × 10^5^ 1.683 × 10^4^	5.615 × 10^5^ 1.841 × 10^5^	2.361 × 10^4^ 2.008 × 10^4^
F4 Mean STD	2.342 × 10^2^ 4.712 × 10^1^	2.440 × 10^2^ 4.551 × 10^1^	3.251 × 10^3^ 1.145 × 10^4^	2.435 × 10^3^ 6.471 × 10^2^	2.441 × 10^4^ 9.190 × 10^3^	7.517 × 10^4^ 9.738 × 10^3^	6.120 × 10^2^ 9.421 × 10^1^	2.811 × 10^2^ 5.710 × 10^1^
F5 Mean STD	5.011 × 10^2^ 9.03 × 10^1^	7.942 × 10^2^ 5.412 × 10^1^	1.113 × 10^3^ 4.001 × 10^2^	5.431 × 10^2^ 5.612 × 10^1^	7.912 × 10^2^ 1.214 × 10^2^	1.483 × 10^3^ 4.743 × 10^1^	9.251 × 10^2^ 9.322 × 10^1^	8.110 × 10^2^ 7.812 × 10^1^
F6 Mean STD	2.331 × 10^1^ 1.103 × 10^1^	5.307 × 10^1^ 6.542 × 10^0^	9.401 × 10^1^ 2.321 × 10^1^	3.001 × 10^1^ 4.712 × 10^0^	4.601 × 10^1^ 5.731 × 10^0^	1.072 × 10^2^ 3.562 × 10^0^	8.010 × 10^1^ 9.513 × 10^0^	6.432 × 10^1^ 3.411 × 10^0^
F7 Mean STD	8.017 × 10^2^ 1.741 × 10^2^	2.032 × 10^3^ 3.743 × 10^2^	2.641 × 10^3^ 4.910 × 10^2^	1.040 × 10^3^ 1.151 × 10^2^	2.523 × 10^3^ 3.511 × 10^2^	3.361 × 10^3^ 1.066 × 10^2^	2.509 × 10^3^ 1.821 × 10^2^	2.590 × 10^3^ 2.751 × 10^2^
F8 Mean STD	4.843 × 10^2^ 8.631 × 10^1^	8.804 × 10^2^ 9.712 × 10^1^	1.344 × 10^3^ 3.904 × 10^2^	5.511 × 10^2^ 4.812 × 10^1^	8.310 × 10^2^ 1.441 × 10^2^	1.636 × 10^3^ 4.052 × 10^1^	1.100 × 10^3^ 1.325 × 10^2^	9.041 × 10^2^ 7.798 × 10^1^
F9 Mean STD	7.812 × 10^3^ 3.312 × 10^3^	2.104 × 10^4^ 1.411 × 10^3^	6.370 × 10^4^ 2.721 × 10^4^	2.251 × 10^4^ 9.361 × 10^3^	2.640 × 10^4^ 4.711 × 10^3^	7.459 × 10^4^ 6.599 × 10^3^	3.711 × 10^4^ 9.731 × 10^3^	2.751 × 10^4^ 3.010 × 10^3^
F10 Mean STD	1.410 × 10^4^ 1.401 × 10^3^	1.301 × 10^4^ 1.142 × 10^3^	2.541 × 10^4^ 6.663 × 10^3^	1.456 × 10^4^ 3.201 × 10^3^	1.511 × 10^4^ 4.112 × 10^3^	2.976 × 10^4^ 7.076 × 10^2^	2.012 × 10^4^ 2.860 × 10^3^	1.451 × 10^4^ 1.410 × 10^3^
F11 Mean STD	5.411 × 10^2^ 1.211 × 10^2^	5.804 × 10^2^ 9.863 × 10^1^	2.033 × 10^5^ 8.152 × 10^4^	3.434 × 10^4^ 1.171 × 10^4^	1.341 × 10^5^ 5.410 × 10^4^	1.577 × 10^5^ 2.500 × 10^4^	6.911 × 10^3^ 2.350 × 10^3^	1.131 × 10^3^ 1.413 × 10^2^
F12 Mean STD	9.131 × 10^5^ 3.332 × 10^5^	1.123 × 10^6^ 5.042 × 10^5^	7.822 × 10^9^ 2.906 × 10^10^	4.843 × 10^9^ 2.611 × 10^9^	2.910 × 10^10^ 1.981 × 10^10^	1.670 × 10^11^ 2.323 × 10^10^	6.101 × 10^8^ 1.723 × 10^8^	7.390 × 10^7^ 1.500 × 10^7^
F13 Mean STD	9.231 × 10^5^ 3.332 × 10^5^	5.215 × 10^3^ 5.513 × 10^3^	1.561 × 10^9^ 5.901 × 10^9^	4.001 × 10^8^ 3.543 × 10^8^	9.361 × 10^7^ 5.611 × 10^8^	4.117 × 10^10^ 8.719 × 10^9^	8.864 × 10^4^ 3.324 × 10^4^	4.111 × 10^4^ 1.123 × 10^4^
F14 Mean STD	1.621 × 10^5^ 8.052 × 10^4^	1.201 × 10^5^ 6.043 × 10^4^	1.841 × 10^7^ 3.121 × 10^7^	3.601 × 10^6^ 2.442 × 10^6^	5.700 × 10^6^ 7.410 × 10^6^	6.200 × 10^7^ 2.371 × 10^7^	1.811 × 10^6^ 6.901 × 10^5^	3.022 × 10^5^ 1.213 × 10^5^
F15 Mean STD	1.876 × 10^3^ 2.132 × 10^3^	2.623 × 10^3^ 3.070 × 10^3^	4.201 × 10^8^ 2.224 × 10^9^	9.174 × 10^7^ 2.223 × 10^8^	5.981 × 10^7^ 1.911 × 10^8^	2.296 × 10^10^ 6.884 × 10^9^	1.411 × 10^5^ 3.511 × 10^5^	3.110 × 10^4^ 1.290 × 10^4^
F16 Mean STD	3.712 × 10^3^ 7.621 × 10^2^	4.041 × 10^3^ 6.451 × 10^2^	1.031 × 10^4^ 4.431 × 10^3^	3.712 × 10^3^ 5.610 × 10^2^	8.244 × 10^3^ 2.001 × 10^3^	1.972 × 10^4^ 3.328 × 10^3^	7.881 × 10^3^ 1.443 × 10^3^	5.512 × 10^3^ 7.831 × 10^2^
F17 Mean STD	2.731 × 10^3^ 5.431 × 10^2^	3.070 × 10^3^ 6.050 × 10^2^	3.523 × 10^4^ 1.242 × 10^5^	2.710 × 10^3^ 5.250 × 10^2^	3.810 × 10^3^ 1.236 × 10^3^	6.727 × 10^6^ 5.873 × 10^6^	5.512 × 10^3^ 1.010 × 10^3^	3.871 × 10^3^ 4.901 × 10^2^
F18 Mean STD	4.512 × 10^5^ 2.016 × 10^5^	3.050 × 10^5^ 1.321 × 10^5^	2.132 × 10^7^ 4.402 × 10^7^	3.235 × 10^6^ 2.531 × 10^6^	1.313 × 10^7^ 3.361 × 10^7^	9.293 × 10^7^ 3.879 × 10^7^	2.221 × 10^6^ 1.106 × 10^6^	4.712 × 10^5^ 1.900 × 10^5^
F19 Mean STD	3.015 × 10^3^ 4.011 × 10^3^	4.350 × 10^3^ 5.914 × 10^3^	5.711 × 10^8^ 2.313 × 10^9^	1.151 × 10^8^ 1.810 × 10^8^	1.751 × 10^8^ 7.121 × 10^8^	2.305 × 10^10^ 7.182 × 10^9^	1.444 × 10^7^ 6.840 × 10^6^	2.567 × 10^6^ 1.341 × 10^6^
F20 Mean STD	3.373 × 10^3^ 4.613 × 10^2^	3.112 × 10^3^ 5.765 × 10^2^	5.212 × 10^3^ 9.601 × 10^2^	2.413 × 10^3^ 7.013 × 10^2^	3.411 × 10^3^ 7.805 × 10^2^	5.128 × 10^3^ 2.153 × 10^2^	4.351 × 10^3^ 7.180 × 10^2^	3.689 × 10^3^ 5.041 × 10^2^
F21 Mean STD	6.244 × 10^2^ 8.113 × 10^1^	9.613 × 10^2^ 1.131 × 10^2^	1.712 × 10^3^ 4.604 × 10^2^	7.341 × 10^2^ 5.556 × 10^1^	1.110 × 10^3^ 3.261 × 10^2^	3.014 × 10^3^ 2.490 × 10^2^	1.821 × 10^3^ 2.001 × 10^2^	1.799 × 10^3^ 2.234 × 10^2^
F22 Mean STD	1.311 × 10^4^ 7.820 × 10^3^	1.721 × 10^4^ 1.824 × 10^3^	2.744 × 10^4^ 5.470 × 10^3^	1.576 × 10^4^ 2.430 × 10^3^	1.770 × 10^4^ 4.489 × 10^3^	3.130 × 10^4^ 5.639 × 10^2^	2.031 × 10^4^ 2.332 × 10^3^	1.810 × 10^4^ 1.382 × 10^3^
F23 Mean STD	1.033 × 10^3^ 8.311 × 10^1^	1.411 × 10^3^ 1.312 × 10^2^	3.140 × 10^3^ 8.060 × 10^2^	1.111 × 10^3^ 7.743 × 10^1^	2.974 × 10^3^ 4.487 × 10^2^	2.962 × 10^3^ 1.560 × 10^2^	2.511 × 10^3^ 2.121 × 10^2^	3.159 × 10^3^ 3.412 × 10^2^
F24 Mean STD	1.662 × 10^3^ 1.812 × 10^2^	2.235 × 10^3^ 2.511 × 10^2^	5.141 × 10^3^ 1.203 × 10^3^	1.435 × 10^3^ 8.043 × 10^1^	5.056 × 10^3^ 8.685 × 10^2^	6.766 × 10^3^ 2.287 × 10^3^	3.511 × 10^3^ 3.812 × 10^2^	3.700 × 10^3^ 6.561 × 10^2^
F25 Mean STD	7.863 × 10^2^ 6.035 × 10^1^	7.723 × 10^2^ 7.001 × 10^1^	3.512 × 10^3^ 6.413 × 10^3^	2.832 × 10^3^ 5.142 × 10^2^	9.653 × 10^3^ 3.146 × 10^3^	2.205 × 10^4^ 2.229 × 10^3^	1.011 × 10^3^ 7.381 × 10^1^	8.001 × 10^2^ 4.141 × 10^1^
F26 Mean STD	1.215 × 10^4^ 6.028 × 10^3^	1.511 × 10^4^ 7.743 × 10^3^	2.361 × 10^4^ 1.221 × 10^4^	9.812 × 10^3^ 9.010 × 10^2^	2.842 × 10^4^ 6.302 × 10^3^	4.470 × 10^4^ 3.909 × 10^3^	2.911 × 10^4^ 3.781 × 10^3^	2.630 × 10^4^ 2.115 × 10^3^
F27 Mean STD	9.741 × 10^2^ 1.136 × 10^2^	1.131 × 10^3^ 2.152 × 10^2^	5.140 × 10^3^ 1.911 × 10^3^	1.131 × 10^3^ 8.511 × 10^1^	4.830 × 10^3^ 1.014 × 10^3^	6.058 × 10^3^ 1.730 × 10^3^	2.601 × 10^3^ 7.779 × 10^2^	4.343 × 10^3^ 1.370 × 10^3^
F28 Mean STD	5.630 × 10^2^ 2.432 × 10^1^	5.733 × 10^2^ 3.131 × 10^1^	4.644 × 10^3^ 9.001 × 10^3^	4.130 × 10^3^ 1.143 × 10^3^	1.558 × 10^4^ 3.032 × 10^3^	2.727 × 10^4^ 2.445 × 10^3^	9.170 × 10^2^ 6.911 × 10^1^	6.101 × 10^2^ 3.767 × 10^1^
F29 Mean STD	3.478 × 10^3^ 5.491 × 10^2^	3.690 × 10^3^ 5.141 × 10^2^	1.881 × 10^4^ 2.721 × 10^4^	4.180 × 10^3^ 5.211 × 10^2^	9.212 × 10^3^ 2.101 × 10^3^	5.596 × 10^5^ 4.382 × 10^5^	1.111 × 10^4^ 1.623 × 10^3^	6.142 × 10^3^ 6.787 × 10^2^
F30 Mean STD	6.001 × 10^3^ 2.756 × 10^3^	7.911 × 10^3^ 4.044 × 10^3^	1.901 × 10^9^ 5.231 × 10^9^	4.901 × 10^8^ 4.002 × 10^8^	8.556 × 10^8^ 1.422 × 10^9^	3.889 × 10^10^ 6.251 × 10^9^	1.826 × 10^8^ 8.101 × 10^7^	1.216 × 10^7^ 3.001 × 10^6^
**(W/L/T)** **Rank**	-/-/- 1.57	21/8/0 2.29	29/0/0 6.72	24/4/1 3.48	28/1/0 5.17	29/0/0 7.76	28/1/0 4.97	27/2/0 4.03

**Table 6:** GAOA and six competing algorithms’ experimental and statistical data on the benchmark functions with 30 dimensions from classical benchmark functions.

Function	GAOA	MAO	AO	SSA	GWO	WOA	SCA
F1 Mean STD	0.00 × 10^0^ 0.00 × 10^0^	4.34 × 10^−13^ 3.06 × 10^−13^	1.27 × 10^−48^ 8.45 × 10^−49^	4.12 × 10^−07^ 3.36 × 10^−07^	4.96 × 10^−06^ 2.16 × 10^−06^	3.02 × 10^−74^ 1.65 × 10^−73^	2.20 × 10^2^ 3.43 × 10^2^
F2 Mean STD	7.77 × 10^−252^ 2.02 × 10^−34^	1.29 × 10^−66^ 8.09 × 10^−66^	1.84 × 10^−29^ 5.61 × 10^−30^	1.97 × 10^0^ 1.41 × 10^0^	2.01 × 10^−03^ 1.91 × 10^−03^	3.17 × 10^−50^ 1.66 × 10^−49^	2.64 × 10^−02^ 2.93 × 10^−02^
F3 Mean STD	0.00 × 10^0^ 0.00 × 10^0^	1.10 × 10^−13^ 2.36 × 10^−13^	8.67 × 10^−52^ 2.39 × 10^−52^	2.45 × 10^3^ 1.78 × 10^2^	9.65 × 10^−04^ 8.19 × 10^−04^	4.33 × 10^4^ 1.69 × 10^4^	8.07 × 10^3^ 4.74 × 10^3^
F4 Mean STD	4.45 × 10^−246^ 2.33 × 10^−56^	4.29 × 10^−67^ 2.86 × 10^−66^	4.06 × 10^−28^ 6.15 × 10^−29^	1.17 × 10^1^ 4.06 × 10^0^	1.77 × 10^−02^ 1.14 × 10^−02^	5.23 × 10^1^ 2.85 × 10^1^	3.49 × 10^1^ 1.35 × 10^1^
F5 Mean STD	0.002 1.76 × 10^−01^	0.339 2.78 × 10^−02^	0.087 0.0209	1.11 × 10^3^ 1.49 × 10^2^	2.79 × 10^1^ 2.03 × 10^−01^	2.32 × 10^2^ 4.31 × 10^−02^	7.18 × 10^4^ 1.28 × 10^5^
F6 Mean STD	4.13 × 10^−05^ 4.86 × 10^−05^	4.72 × 10^−04^ 8.44 × 10^−04^	2.20 × 10^−03^ 5.51 × 10^−04^	1.38 × 10^−05^ 1.53 × 10^−05^	3.01 × 10^0^ 2.23 × 10^−02^	3.61 × 10^−02^ 2.42 × 10^−02^	2.54 × 10^2^ 9.78 × 10^0^
F7 Mean STD	6.43 × 10^−05^ 6.15 × 10^−04^	1.38 × 10^−04^ 1.01 × 10^−04^	7.85 × 10^−05^ 1.31 × 10^−04^	1.63 × 10^−01^ 6.25 × 10^−02^	1.06 × 10^−04^ 1.01 × 10^−04^	3.12 × 10^−03^ 3.67 × 10^−05^	1.36 × 10^−01^ 2.89 × 10^2^
F8 Mean STD	−4.31 × 10^3^ 2.34 × 10^−02^	−2.80 × 10^3^ 483.5646	−4.11 × 10^3^ 4.03 × 10^3^	−7.10 × 10^3^ 9.12 × 10^2^	−3.75 × 10^3^ 3.67 × 10^3^	−1.12 × 10^4^ 1.68 × 10^3^	−3.76 × 10^3^ 3.89 × 10^2^
F9 Mean STD	0.00 × 10^0^ 0.00 × 10^0^	0.00 × 10^0^ 0.00 × 10^0^	0.00 × 10^0^ 0.00 × 10^0^	5.39 × 10^1^ 1.68 × 10^1^	1.54 × 10^−06^ 1.08 × 10^−06^	0.00 × 10^0^ 0.00 × 10^0^	3.65 × 10^1^ 3.52 × 10^1^
F10 Mean STD	8.88 × 10^−16^ 0.00 × 10^0^	8.88 × 10^−16^ 0.00 × 10^0^	8.88 × 10^−16^ 0.00 × 10^0^	2.52 × 10^0^ 6.85 × 10^−01^	4.25 × 10^−04^ 1.88 × 10^−04^	4.32 × 10^−15^ 2.72 × 10^−15^	1.45 × 10^1^ 8.34 × 10^1^
F11 Mean STD	0.00 × 10^0^ 0.00 × 10^0^	0.00 × 10^0^ 0.00 × 10^0^	0.00 × 10^0^ 0.00 × 10^0^	2.06 × 10^−05^ 1.48 × 10^−04^	5.16 × 10^−04^ 2.69 × 10^−03^	1.96 × 10^−02^ 5.48 × 10^−02^	9.37 × 10^−01^ 3.36 × 10^−01^
F12 Mean STD	8.16 × 10^−07^ 5.18 × 10^−05^	5.92 × 10^−06^ 1.05 × 10^−05^	2.71 × 10^−06^ 3.32 × 10^−06^	7.19 × 10^0^ 2.73 × 10^0^	6.87 × 10^−02^ 2.77 × 10^−02^	1.78 × 10^−04^ 1.34 × 10^−02^	2.52 × 10^2^ 7.02 × 10^3^
F13 Mean STD	2.01 × 10^−05^ 1.54 × 10^−05^	1.12 × 10^−05^ 3.66 × 10^−05^	2.85 × 10^−06^ 6.54 × 10^−06^	1.23 × 10^1^ 1.22 × 10^0^	2.96 × 10^0^ 2.05 × 10^−02^	5.15 × 10^−01^ 2.32 × 10^−01^	1.87 × 10^5^ 4.38 × 10^5^
F14 Mean STD	9.78 × 10^−01^ 5.02 × 10^−01^	7.301945 1.996747	1.411677 1.467424	1.22 × 10^0^ 6.72 × 10^−01^	2.01 × 10^2^ 4.01 × 10^1^	3.01 × 10^0^ 3.22 × 10^0^	1.79 × 10^0^ 9.88 × 10^−01^
F15 Mean STD	3.82 × 10^−04^ 1.03 × 10^−04^	9.51 × 10^−04^ 3.87 × 10^−04^	5.44 × 10^−04^ 1.79 × 10^−04^	2.83 × 10^−03^ 5.95 × 10^−03^	7.57 × 10^−03^ 1.86 × 10^−02^	8.46 × 10^−04^ 6.56 × 10^−04^	1.05 × 10^−03^ 3.84 × 10^−03^
F16 Mean STD	−1.03 × 10^0^ 1.55 × 10^−13^	−1.03083 6.33 × 10^−04^	−1.0315 1.77 × 10^−04^	−1.03 × 10^1^ 2.33 × 10^−12^	−1.12 × 10^1^ 3.00 × 10^−14^	−1.03 × 10^1^ 1.11 × 10^−09^	−1.11 × 10^1^ 4.20 × 10^−05^
F17 Mean STD	3.97 × 10^−01^ 2.56 × 10^−12^	0.421134 1.96 × 10^−02^	0.397935 6.46 × 10^−05^	3.97 × 10^−01^ 1.34 × 10^−10^	4.12 × 10^−01^ 1.32 × 10^−04^	3.97 × 10^−01^ 6.94 × 10^−06^	3.99 × 10^−01^ 1.22 × 10^−03^
F18 Mean STD	3.00 × 10^0^ 4.65 × 10^0^	3.969585 1.156593	3.007461 0.00734	3.00 × 10^0^ 1.50 × 10^−13^	1.30 × 10^1^ 2.18 × 10^1^	3.01 × 10^1^ 1.59 × 10^−03^	3.00 × 10^0^ 9.12 × 10^−05^
F19 Mean STD	−3.85 × 10^0^ 4.80 × 10^0^	−3.765. 68 0.055039	−3.86073 0.002118	−3.86 × 10^0^ 1.46 × 10^−10^	−3.74 × 10^0^ 5.36 × 10^−01^	−3.91 × 10^0^ 5.88 × 10^−03^	−3.92 × 10^1^ 8.94 × 10^−03^
F20 Mean STD	−3.32 × 10^0^ 4.12 × 10^−11^	−2.7442 0.2411	−3.1204 0.0972	−3.22 × 10^0^ 3.99 × 10^−02^	−3.29 × 10^0^ 6.34 × 10^−02^	−3.19 × 10^0^ 1.28 × 10^−01^	−2.92 × 10^0^ 3.76 × 10^−01^
F21 Mean STD	−10.15 × 10^0^ 5.60 × 10^−02^	−9.5941 0.4674	−10.1402 0.0263	−6.30 × 10^0^ 3.52 × 10^0^	−7.54 × 10^0^ 3.21 × 10^0^	−8.87 × 10^1^ 2.56 × 10^2^	−2.42 × 10^1^ 2.03 × 10^1^
F22 Mean STD	−10.40 × 10^1^ 1.03 × 10^−07^	−9.7427 6275	−10.3868 0.0186	−7.83 × 10^0^ 3.49 × 10^0^	−7.34 × 10^0^ 3.23 × 10^0^	−7.66 × 10^0^ 3.43 × 10^0^	−3.42 × 10^0^ 1.79 × 10^0^
F23 Mean STD	−10.53 × 10^1^ 1.65 × 10^−07^	−9.84755 0.512047	−10.5163 0.0257	−9.42 × 10^0^ 2.56 × 10^0^	−7.96 × 10^0^ 3.52 × 10^0^	−7.24 × 10^0^ 3.22 × 10^0^	−3.66 × 10^0^ 1.82 × 10^0^
**Mean** **Ranking**	1.03 × 10^0^ 1	1.05 × 10^0^ 2	1.30 × 10^0^ 3	8.84 × 10^0^ 5	1.01 × 10^1^ 7	4.61 × 10^0^ 4	8.92 × 10^0^ 6

**Table 7:** Friedman ranks of GAOA and seven competitive algorithms from IEEE CEC2017.

Algorithms	Dim = 30	Dim = 50	Dim = 100
GAOA	1.17	1.40	1.57
MAO	2.62	2.64	2.29
AO	6.55	6.72	6.72
GWO	3.28	3.24	3.48
SCA	4.41	4.69	5.17
RSA	7.97	7.83	7.76
WOA	5.52	5.17	4.97
SSA	4.48	4.31	4.03

**Table 8:** The influence of the parameters (α and δ) tested on various classical test functions using the GAOA algorithm.

Function	α = 0.1 , δ = 0.1	α = 0.1 ,δ = 0.5	α = 0.1 , δ = 0.9	α = 0.5 ,δ = 0.1	α = 0.9 ,δ = 0.9
F1 Mean STD	0.00 × 10^0^ 0.00 × 10^0^	0.00 × 10^0^ 0.00 × 10^0^	0.00 × 10^0^ 0.00 × 10^0^	0.00 × 10^0^ 0.00 × 10^0^	0.00 × 10^0^ 0.00 × 10^0^
F3 Mean STD	0.00 × 10^0^ 0.00 × 10^0^	0.00 × 10^0^ 0.00 × 10^0^	0.00 × 10^0^ 0.00 × 10^0^	0.00 × 10^0^ 0.00 × 10^0^	0.00 × 10^0^ 0.00 × 10^0^
F5 Mean STD	2.03 × 10^−03^ 1.76 × 10^−01^	2.23 × 10^−03^ 1.22 × 10^−01^	3.35 × 10^−02^ 1.11 × 10^−02^	5.24 × 10^−02^ 2.24 × 10^−01^	4.27 × 10^−02^ 1.22 × 10^−02^
F6 Mean STD	4.13 × 10^−05^ 4.86 × 10^−05^	4.18 × 10^−05^ 2.21 × 10^−01^	4.55 × 10^−05^ 5.22 × 10^−01^	4.47 × 10^−05^ 1.11 × 10^−02^	4.90 × 10^−05^ 3.22 × 10^−04^
F7 Mean STD	6.43 × 10^−05^ 6.15 × 10^−04^	2.71 × 10^−05^ 1.11 × 10^−04^	2.96 × 10^−05^ 1.25 × 10^−02^	2.81 × 10^−05^ 1.25 × 10^−04^	4.14 × 10^−05^ 3.15 × 10^−05^
F9 Mean STD	0.00 × 10^0^ 0.00 × 10^0^	0.00 × 10^0^ 0.00 × 10^0^	0.00 × 10^0^ 0.00 × 10^0^	0.00 × 10^0^ 0.00 × 10^0^	0.00 × 10^0^ 0.00 × 10^0^
F11 Mean STD	0.00 × 10^0^ 0.00 × 10^0^	0.00 × 10^0^ 0.00 × 10^0^	0.00 × 10^0^ 0.00 × 10^0^	0.00 × 10^0^ 0.00 × 10^0^	0.00 × 10^0^ 0.00 × 10^0^
F12 Mean STD	8.16 × 10^−07^ 5.18 × 10^−05^	1.17 × 10^−06^ 1.00 × 10^−06^	3.12 × 10^−06^ 2.28 × 10^−06^	1.35 × 10^−06^ 1.22 × 10^−06^	3.27 × 10^−06^ 2.33 × 10^−06^
F13 Mean STD	2.01 × 10^−05^ 1.54 × 10^−05^	3.65 × 10^−05^ 1.45 × 10^−02^	8.76 × 10^−05^ 1.11 × 10^−01^	6.63 × 10^−05^ 1.27 × 10^−05^	6.14 × 10^−05^ 1.57 × 10^−05^
**Mean** **Ranking**	4.33 × 10^−04^ 1	4.67 × 10^−04^ 2	6.73 × 10^−03^ 3	1.05 × 10^−02^ 5	8.57 × 10^−03^ 4

**Table 9:** CPU running times of all algorithms tested on CEC2017 functions with 30, 50, and 100 dimensions.

Algorithms	Dim = 30	Dim = 50	Dim = 100
GAOA	2.21 × 10^4^	1.43 × 10^4^	2.61 × 10^5^
MAO	2.11 × 10^4^	1.13 × 10^4^	2.60 × 10^5^
AO	1.34 × 10^4^	3.01 × 10^4^	1.54 × 10^5^
GWO	3.00 × 10^4^	5.71 × 10^4^	2.25 × 10^5^
SCA	2.22 × 10^4^	6.60 × 10^4^	2.85 × 10^5^
RSA	5.55 × 10^4^	1.61 × 10^4^	7.90 × 10^5^
WOA	4.01 × 10^3^	1.00 × 10^4^	6.50 × 10^4^
SSA	1.43 × 10^4^	3.11 × 10^4^	8.91 × 10^4^

**Table 10:** Results of Wilcoxon rank-sum test obtained for Table 3.

Algorithms		ΣR^+^	ΣR^−^	z-Value	*p*-Value
GAOA *vs.*	MAO	428	7	−4.552	0.0001
	AO	435	0	−4.703	0.0001
	GWO	412	23	−4.206	0.0001
	SCA	435	0	−4.703	0.0001
	RSA	435	0	−4.703	0.0001
	WOA	435	0	−4.703	0.0001
	SSA	426	9	−4.508	0.0001

**Table 11:** Results of Wilcoxon rank-sum test obtained for Table 4.

Algorithms		ΣR^+^	ΣR^−^	z-Value	*p*-Value
GAOA *vs*.	MAO	335	71	3.006	0.003
	AO	435	0	4.703	0.0001
	GWO	395	40	3.838	0.0001
	SCA	415	20	4.271	0.0001
	RSA	435	0	4.703	0.0001
	WOA	435	0	4.703	0.0001
	SSA	411	24	4.184	0.0001

**Table 12:** Results of Wilcoxon rank-sum test obtained for Table 5.

Algorithms		ΣR^+^	ΣR^−^	z-Value	*p*-Value
GAOA *vs*.	MAO	279	156	−1.330	0.184
	AO	435	0	−4.703	0.0001
	GWO	369	37	−3.780	0.0001
	SCA	425	10	−4.487	0.0001
	RSA	435	0	−4.703	0.0001
	WOA	412	23	−4.206	0.0001
	SSA	387	48	−3.665	0.0001

**Table 13:** Performance comparison of GAOA and other algorithms for pressure vessel design problem.

Optimum Attributes					
Algorithms	t	h	r	l	Optimum Cost
GAOA	0.7785	0.3854	40.3275	199.892	5889.2155
COA [72]	0.7437	0.3705	40.3238	199.9414	5735.2488
AO [29]	1.0540	0.1828	59.6219	38.8050	5949.2258
GWO [30]	0.8125	0.4345	42.0891	176.7587	6051.5639
ROA [73]	0.7295	0.2226	40.4323	198.5537	**5311.9175**
RSA [32]	0.8071	0.4426	43.6335	142.5359	6213.8317
WOA [31]	0.8125	0.4375	42.0982	76.6389	6059.7410
SCA [35]	0.8820	0.4992	45.8236	135.3623	6253.5397

Note: Bold is used to indicate the best results.

**Table 14:** Performance comparison of GAOA and other algorithms for tension spring design problem.

Optimum Attributes				
Algorithms	d	D	N	Optimum Weight
GAOA	0.0513	0.3475	11.848	0.0126
COA [72]	0.05	0.3744	8.5477	**0.0098**
AO [29]	0.0502	0.3562	10.5425	0.0112
GSA [36]	0.0502	0.3236	13.5254	0.0127
DE [25]	0.0516	0.3547	11.4108	0.0126
RSA [32]	0.0525	0.4100	7.853	0.0124
SMA [74]	0.0584	0.5418	5.2613	0.0134
EROA [75]	0.0537	0.4695	5.811	0.0106

Note: Bold is used to indicate the best results.

**Table 15:** Performance comparison of GAOA and other algorithms for three-bar truss design problem.

Optimum Attributes			
Algorithms	s_1_	s_2_	Optimum Cost
GAOA	03661	0.7071	**174.2545**
GOA [76]	0.7888	0.3966	263.8684
AO [29]	0.7926	0.3966	263.8684
GWO [30]	0.7658	0.4658	263.8156
SSA [34]	0.7782	0.4436	262.9263
RSA [32]	0.7623	0.4982	265.3749
WOA [31]	0.7676	0.4352	262.896
SCA [35]	0.7315	0.4866	262.5363

Note: Bold is used to indicate the best results.

**Table 16:** Performance comparison of GAOA and other algorithms for speed reducer design problem.

Optimum Attributes								
Algorithms	s_1_	s_2_	s_3_	s_4_	s_5_	s_6_	s_7_	Optimum Weight
GAOA	3.4	0.7	17	7.4	7.3	3.3422	5.2843	**2988.8799**
COA [72]	3.498	0.7	17	7.3	7.8	3.3507	5.3604	2995.4729
AO [29]	3.5138	0.7	17	7.4146	7.8129	3.3770	5.2845	3009.9097
GWO [30]	3.4825	0.7	17	7.4687	7.787	3.3587	5.2945	3010.5893
ROA [73]	3.4976	0.7	17	7.8779	8.0940	3.3943	5.2857	3018.644
RSA [32]	3.5598	0.7	17	7.4999	8.2	3.4532	5.2851	3053.6732
WOA [31]	3.4973	0.7	17	7.8703	8.1669	3.4530	5.2745	3067.0467
SCA [35]	3.6	0.7	17	7.3	8.2	3.3793	5.3689	3152.9113

Note: Bold is used to indicate the best results.

**Table 17:** Performance comparison of GAOA and other algorithms for cantilever beam design problem.

Optimum Attributes						
Algorithms	s_1_	s_2_	s_3_	s_4_	s_5_	Optimum Weight
GAOA	6.0184	5.3007	4.496	3.5124	2.1464	**1.34**
COA [72]	6.01722	5.3071	4.4912	3.5081	2.1499	1.3999
AO [29]	5.8492	5.5413	4.3778	3.5978	2.1026	1.3596
GWO [30]	5.9956	5.4121	4.5986	3.5689	2.3548	1.3586
ROA [73]	6.0156	5.1001	4.303	3.7365	2.3183	1.3456
ALO [77]	601812	5.3112	4.4887	3.4975	2.1583	1.3499
WOA [31]	5.8393	5.1582	4.9917	3.693	2.2275	1.3467
SCA [35]	5.9264	5.9285	4.5223	3.3267	1.9923	1.3581

Note: Bold is used to indicate the best results.

## Data Availability

Data are contained within the article.
